# New stable QTLs for berry weight do not colocalize with QTLs for seed traits in cultivated grapevine (*Vitis vinifera* L.)

**DOI:** 10.1186/1471-2229-13-217

**Published:** 2013-12-19

**Authors:** Agnès Doligez, Yves Bertrand, Marc Farnos, Michel Grolier, Charles Romieu, Florence Esnault, Sonia Dias, Gilles Berger, Pierre François, Thierry Pons, Patrick Ortigosa, Catherine Roux, Cléa Houel, Valérie Laucou, Roberto Bacilieri, Jean-Pierre Péros, Patrice This

**Affiliations:** 1 INRA, UMR AGAP, Batiment 21 2 place Viala, F-34060 Montpellier Cedex 1, France

**Keywords:** Berry weight, Candidate gene, Grapevine, Quantitative trait locus, QTL, Seed number, Seed weight, *Vitis vinifera*

## Abstract

**Background:**

In grapevine, as in other fruit crops, fruit size and seed content are key components of yield and quality; however, very few Quantitative Trait Loci (QTLs) for berry weight and seed content (number, weight, and dry matter percentage) have been discovered so far. To identify new stable QTLs for marker-assisted selection and candidate gene identification, we performed simultaneous QTL detection in four mapping populations (seeded or seedless) with various genetic backgrounds.

**Results:**

For berry weight, we identified five new QTLs, on linkage groups (LGs) 1, 8, 11, 17 and 18, in addition to the known major QTL on LG 18. The QTL with the largest effect explained up to 31% of total variance and was found in two genetically distant populations on LG 17, where it colocalized with a published putative domestication locus. For seed traits, besides the major QTLs on LG 18 previously reported, we found four new QTLs explaining up to 51% of total variance, on LGs 4, 5, 12 and 14. The previously published QTL for seed number on LG 2 was found related in fact to sex. We found colocalizations between seed and berry weight QTLs only for the major QTL on LG 18 in a seedless background, and on LGs 1 and 13 in a seeded background. Candidate genes belonging to the cell number regulator CNR or cytochrome P450 families were found under the berry weight QTLs on LGs 1, 8, and 17. The involvement of these gene families in fruit weight was first described in tomato using a QTL-cloning approach. Several other interesting candidate genes related to cell wall modifications, water import, auxin and ethylene signalling, transcription control, or organ identity were also found under berry weight QTLs.

**Conclusion:**

We discovered a total of nine new QTLs for berry weight or seed traits in grapevine, thereby increasing more than twofold the number of reliable QTLs for these traits available for marker assisted selection or candidate gene studies. The lack of colocalization between berry and seed QTLs suggests that these traits may be partly dissociated.

## Background

Fruit size is a major determinant of both yield and quality for many crops with fleshy fruits. A comprehensive understanding of its genetic determinism is crucial to elucidate fruit development mechanisms and facilitate the breeding of new varieties. Although the genetic architecture of fruit size has been investigated in several species (e.g. tomato, peach, grape, apple, cherry, melon, citrus, papaya, cranberry), genes underlying Quantitative Trait Loci (QTLs) have been so far identified in tomato only. Grapevine appears as a particularly significant model to study fruit development, since it is the only non-climacteric fruit among the few fruit crop species almost fully sequenced [1,2].

Berry size and seed content are essential selection criteria in grape breeding. Berry size and number are major yield components for both table fruit and wine production. However, large berries are desirable only for table grape while smaller fruits are preferred for winemaking. In wine cultivars, small berry size is principally searched for to increase skin-to-flesh ratio, thus improving final concentrations of anthocyanins, tannins and aroma compounds in wine. These chemicals are primarily localized in the skin, but seeds are another important source of condensed tannins. Conversely in table cultivars, reduced seed perceptibility and large berries are sought. Therefore, the possible physiological correlation between berry size and seed quantity is a major issue in grape breeding.

Large variation in berry and seed traits has been observed among cultivars of the cultivated grapevine *Vitis vinifera* L. On average, berry fresh weight at maturity ranges from 0.5 to 11 g [3], seed number from 0 to 3.6 and seed weight per berry from 0.03 to 0.22 g [4].

Similarly to other fleshy fruits [5], grapevine pericarp grows from fertilization to maturity according to a double sigmoid curve, with three main development stages [6-8]. Stage I involves both cell division and enlargement through the accumulation into vacuoles of water and organic acids. During stage II, berry growth slows down or stops. At the onset of the following growth period (stage III), referred to as *véraison*, many rapid physiological changes occur, among which berry softening and skin coloring in red cultivars. Berry size roughly doubles during this ripening phase, as a result of vacuolar expansion triggered by water and sugars accumulation in the mesocarp (flesh). Maximal berry size depends on both cell number and size [5,7] and is largely defined already at *véraison*[9]. Berry growth is under the control of several growth regulators: ethylene [10,11], auxins and ABA [12], and gibberellins [13,14].

Normal seed development from fertilization to maturity involves the three following phases: i) rapid cell division; ii) reserve accumulation and cell expansion due to water uptake; iii) slowing down and then arrest of reserve accumulation [15]. In the case of stenospermocarpic seedlessness, which originates from cv. Sultanina, intensively used as a genitor in table grape breeding, the ovule is successfully fertilized, embryo and endosperm cell divisions begin, but then the endosperm degenerates at various stages. Seeds do not achieve full development, although embryos are viable and can develop into new plants [16]. Stenospermocarpic seedlessness can therefore be described with several quantitative traits (seed number, fresh and dry weight, seed coat hardness, endosperm development) [17], some of which will also vary in seeded grapes.

The widely accepted correlation between berry weight and seed content is not so evident. A positive correlation between berry final weight and seed content has been frequently observed within seeded cultivars [8,18-23], within populations segregating for seedlessness [24-30] and in pools composed of both seeded and seedless table grape breeding populations [31,32]. By contrast, no significant correlation was found between seed content and berry size within a set of 190 new table grape cultivars [33] or in a set of 254 highly diverse cultivars [4]. It is generally admitted that the correlation between berry size and seed content mainly results from growth regulators produced by the seeds [16,34,35]. However, some authors found that exogenous gibberellins had no effect on berry size in seeded cultivars [34,36,37].

Despite high heritabilities, the genetic determinism of berry weight and seed content variation is far less documented than the effect of environmental factors. Heritability of berry weight and seedlessness seems relatively high and mainly additive. Indeed, reported values for narrow-sense heritability were 0.63-0.69 for berry weight [31,32,38] and 0.58 for seedlessness [31]. Similarly, broad-sense heritability values were 0.49-0.92 for berry weight [39-42], 0.996 for total seed weight per berry [39], and 0.34 for seed number [39]. All previously published QTL studies in a stenospermocarpic seedless background found a major QTL for both berry weight and seed traits on LG 18 [25,27-29]. The most probable candidate gene for this QTL is the *VvAGL11* gene, an ortholog of a MADS-box gene involved in ovule differentiation in *Arabidopsis* and petunia [30]. For berry size, only two QTLs stable over time were found in addition to the above QTL on LG18 [43], the other ones being unstable (Additional file 1: Table S1). For seed traits, four stable minor QTLs were reported in addition to the major QTL on LG 18. Houel et al. [44,45] found five SNPs associated to berry weight. Despite all these efforts, no gene involved in berry size variation has been identified so far.

The objectives of this study were: i) to find QTLs for berry weight and seed traits stable enough to be used in Marker Assisted Selection and for searching for candidate genes harboring causal mutations, ii) to assess the extent of seed and berry QTLs colocalization, and iii) to propose positional candidate genes for further evaluation. To achieve these goals, we performed simultaneous QTL detection over several years in various genetic backgrounds (two seedless and two seeded pseudo-F1 families). This allowed us to discover five new stable QTLs for berry weight and four new stable QTLs for seed traits, with little colocalization between berry and seed QTLs, and to propose a few promising candidate genes in these regions.

## Methods

### Plant material

This study was based on four pseudo-F1 mapping populations (Table 1). MTP3140 and MTP3234 populations were over-grafted in 1993 [46] and 2001-2002 [47], respectively, at the INRA Chapitre experimental station, Hérault, France. A population obtained from a cross between cv. Syrah and cv. Grenache (SxG) was grafted and planted in two complete randomized blocks in 2003 at the same experimental station. MTP3346 progeny plants were grown in a greenhouse at the CTIFL experimental station of Balandran, Gard, in 2001-2003. In 2004, a random subset of fertile plants from this population were installed outside in containers at the Chapitre experimental station, without replication.

**Table 1:** Progenies and experimental designs used for QTL detection in four grapevine mapping populations

**Population**	**Parents**	**Nb. offsprings**	**Genetic background**	**Nb. replicates**	**Nb. plants / offspring**	**Years of harvest and phenotyping**	**Nb.clusters harvested per offspring**	**Reference of genetic map**
MTP3140	MTP2223-27 (Dattier de Beyrouth × 75 Pirovano)	139	table seedless	1	1	1994^4^, 1995^4^, 1996^4^, 1998, 1999	all	[48]
	x MTP2121-30 (Alphonse Lavallée × Sultanine)							
MTP3234	MTP2687-85 (Olivette noire × Ribol)	174	table seeded	1	1	2002^5^, 2003, 2004	all	[47]
	x Muscat of Hamburg)							
SxG^1, 2^	Grenache × Syrah	96	wine seeded	2	5	2005^6^, 2006, 2007	8	modified from [49]^8^
	Syrah × Grenache	95						
MTP3346^3^	Muscat of Alexandria	519	table seedless	1	1	2003, 2005^7^	all	[48]
	x MTP3140-517 (selected from the MTP3140 population)							

^1^Reciprocal cross.

^2^Seed dry weight not evaluated in SxG.

^3^Partial QTL detection in ten genomic regions only.

^4^Phenotypic data already available from [46] and [25], with only slight differences in phenotyping protocol.

^5^Seed traits not evaluated in 2002.

^6^Phenotyping of one replicate only in 2005.

^7^Phenotyping on a sample of 142 individuals in 2005.

^8^Maps of Huang et al. [49] but based on the Kosambi (instead of Haldane) mapping function.

### Phenotyping

Clusters of each fruit-bearing offspring were harvested at maturity during at least two years (Table 1). End parts of clusters were discarded and 100 berries randomly sampled and weighted to estimate mean berry weight (MBW). Seeds were extracted from 25 random berries to determine mean seed number per berry (MSN), total seed fresh weight per berry (TSFW), mean seed fresh weight (MSFW) and the percentage of seed dry matter after drying at 80°C for 72 hours (%SDM). The residuals obtained by linear regression using SAS/STAT® software v9.1.3 (SAS Institute Inc., Cary, NC) of MBW on either MSN or TSFW (RESN and RESFW, respectively) were used as additional traits for QTL analyses.

### Normality, genetic values and heritability

Statistical analyses were performed on raw phenotypic data from each year using the R statistical base and pgirmess packages [50]. Distribution normality was evaluated using the Shapiro-Wilk test [51]. Since most data distributions significantly deviated from normality, we used non-parametric procedures to calculate and test phenotypic correlations (Spearman rank-order correlation coefficient).

When data distribution deviated from normality, we applied either square root (sqrt) or neperian logarithm (ln) transformation to unskew distribution, after adding 1, 2 or 3 to the raw data to obtain positive values. These transformed values were used to estimate the Best Linear Unbiased Predictors (BLUP) of genetic values across blocks and/or years with SAS/STAT, for use in QTL detection. Models selected were those with the lowest Bayesian Information Criterion (BIC), among several models always including a random genotypic effect, completed or not by a fixed year and/or block effect. For the SxG population, the full model was: *P*_*ijk*_ = *μ* + *G*_*i*_ + *b*_*j*_ + *y*_*k*_ + *e*_*ijk*_, where *P*_*ijk*_ was the phenotypic value of genotype *i* in block *j* and year *k*, *μ* the overall mean, *G*_*i*_ the random effect of genotype *i*, *b*_*j*_ the fixed effect of block *j*, *y*_*k*_ the fixed effect of year *k* and *e*_*ijk*_ the residual error effect. Genetic correlations (Spearman’s test) were estimated between BLUPs. Variance estimates of the selected models were used to estimate broad-sense heritabilities on an inter-annual genotype mean basis, defined as $H^{2}=\sigma_{G}^{2}/\left(\sigma_{G}^{2}+\left(\sigma_{e}^{2}/n\right)\right),$ where $\sigma_{G}^{2}$ and $\sigma_{e}^{2}$ were the genotypic and residual variances, respectively, and *n* the mean number of replicates (*n* = 1 when year effect was significant, otherwise *n* = mean number of replicates with non-missing data).

### QTL detection

Parental and consensus genetic maps were already available (Table 1). Since only a partial genetic map was available for population MTP3346 (10 genomic regions spanning less than a quarter of the genome), the results for this population were provided as additional files and only mentioned to complement the other results.

A summary of map features is given in Additional file 2: Table S2). QTL detection was performed on parental and consensus maps as follows. Composite interval mapping (CIM) was applied to parental maps using WinQTLCart 2.0 [52], with cofactors selected through forward and backward (FB) regressions. LOD thresholds corresponding to a genome-wide type I error rate of α = 5% were determined using 1,000 permutations of traits over marker data, with cofactors reselected for each permuted data set, using QTLCartographer 1.17 [53,54].

MQM (Multiple QTL Model, a CIM equivalent) was performed on consensus maps using MapQTL 4.0 [55], with cofactors selected as the markers nearest to the QTLs detected with interval mapping (IM) and which passed the MapQTL automatic cofactor selection procedure (backward elimination). The *α* = 5% genome-wide LOD thresholds were those obtained for IM with 1,000 permutations, empirically corrected by adding 0.5 to take into account higher threshold values in CIM compared to IM. To check dubious QTLs (*e.g.* in case of distorted segregation, large interval between nearest markers, or skewed distribution of residuals), the CIM results were complemented by a non-parametric Kruskal-Wallis rank sum test (for the consensus map) or variance analysis (for parental maps). Confidence intervals of QTL positions were defined as one-LOD support intervals. QTL detection was carried out with the transformed phenotypic data for each year (BLUPs of genetic values across blocks for SxG in 2006 and 2007) to check QTL stability over time, but also on the BLUPs of genetic values across years. For QTLs detected on consensus maps, female, male and dominance allelic effects were estimated according to [56], as *Af* = [(*μad* + *μac*)-(*μbd* + *μbc*)]/4, *Am* = [(*μac* + *μbc*)-(*μad* + *μbd*)]/4 and *D* = [(*μac* + *μbd*)-(*μbc* + *μad*)]/4, respectively, where *μac*, *μad*, *μbc* and *μbd* were the phenotypic means estimated for each of the four possible genotypic classes, *ac*, *ad*, *bc* and *bd*, respectively, in the progeny of an *ab* × *cd* cross.

### Candidate genes

To identify the most probable candidate genes, we used a double approach. First, consulting the available literature or databases, we established a list of genes possibly involved in berry weight according to their putative function (*e.g.* cell replication, water transport, or cell wall metabolism), as well as to the role of their homologue on fruit weight in other species or to published results obtained for expression, transformation or association genetics in grapevine. We then checked whether these candidate genes colocalized with the detected QTLs in our mapping populations. In particular, we explored two gene families involved in fruit weight QTLs cloned in tomato, the CNR and P450 78A families [57,58]. To search for homology and genome position, we used the BlastP algorithm to match the proteic sequence against the NCBI *Vitis* refseq_protein database.

Secondly, as a complementary approach, we looked for the limited number of genes with a putative function relevant to berry weight, within the numerous genes present under the QTLs, even in the absence of published results in grapevine or cloned QTLs in other fleshy fruit species. Taking into account a review of fine mapped QTLs [59], showing that causal polymorphisms were found within 3 cM of LOD peaks, we extracted positional candidate genes from a +/- 3 cM interval around the LOD peak of each stable BLUP QTL for berry weight on consensus maps. The physical interval limits were extrapolated from the positions of flanking SSR markers. Accession numbers, positions and putative functions of predicted mRNA were extracted from ref_12X_top_level.gff3.gz, last modification 03/15/2012 downloaded at ftp://ftp.ncbi.nih.gov/genomes/Vitis_vinifera/GFF/, obtained with the GNOMON method (http://www.ncbi.nlm.nih.gov/genome/guide/gnomon.shtml). We then checked in QTL intervals the presence of functional candidate genes.

## Results

### Phenotypic data

Raw measurement distributions were very similar between years but showed differences between populations (Additional file 3: Figure S1). The extent of variation in berry weight was larger for the three table grape populations than for the SxG wine grape population, whereas it was more similar among populations for seed number, despite the large zero class in the seedless MTP3140 population. Seed weights presented more variation in MTP3140 and MTP3234 than in SxG and MTP3346.

All traits showed continuous variation and transgressive segregation. Regression of MBW on MSN or TSFW was always highly significant, except for MSN in SxG in 2006 and 2007. Deviation from normality was observed for most traits in most populations and appropriate transformations were therefore applied (Additional file 4: Table S3). Phenotypic correlation between years varied from 0.38 to 0.97 depending on trait and population (Additional file 5: Table S4) and was always highly significant.

**Figure 1:**
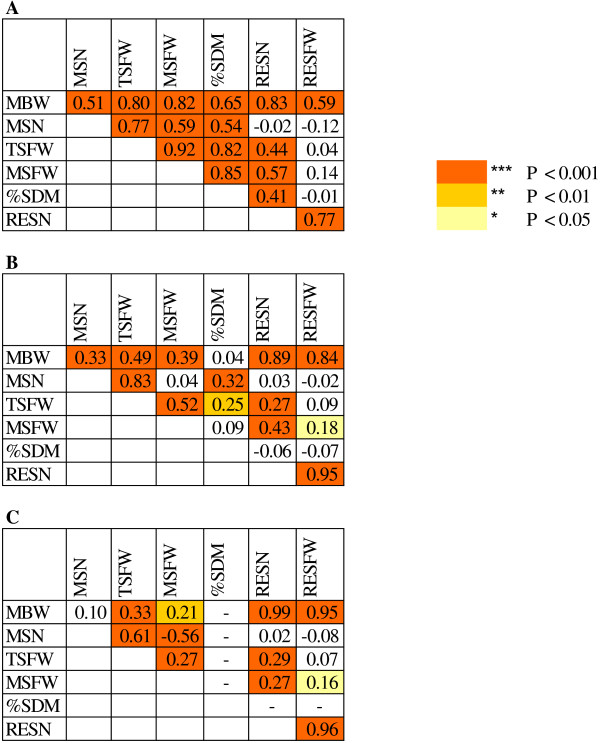
**Genetic correlations between seven seed and berry-related traits within years in three grapevine mapping populations.** Genetic correlations among BLUPs of the genetic value for the populations MTP3140 (A), MTP3234 (B), SxG (C). Background cell color indicates Spearman test significance: α=5% (light yellow), 1% (dark yellow), 0.1% (orange), not significant (white). MBW: mean berry weight; MSN: mean seed number; TSFW: total seed fresh weight; MSFW: mean seed fresh weight; %SDM: seed dry matter percentage; RESN: residual berry weight unexplained by seed number; RESFW: residual berry weight unexplained by total seed fresh weight.

Most of the models selected to estimate BLUPs of genetic values and heritabilities included year effect (Additional file 4: Table S3). In SxG population, selected models never included block effect (for both within and among year models). Genetic correlation between traits are shown in Figure 1 and Additional file 6: Figure S2). Berry weight was highly significantly correlated with total seed fresh weight in all populations (*P* < 0.001), the correlation value varying between 0.29 and 0.80. Correlation values between berry weight and seed number were lower (0.10-0.51) and not always significant (*P* > 0.05 for SxG). RESN and RESFW were actually uncorrelated to seed number and total fresh weight, as expected, whereas they remained tightly correlated with berry weight (0.59-0.99) and between each other (0.77-0.96). The correlation between seed number and mean seed weight was significantly positive in the two seedless populations but not significant or significantly negative in the two seeded populations.

Broad sense heritability of the interannual genotypic means was rather large and varied between 0.51 and 0.95 (Table 2 and Additional file 7: Table S5).

**Table 2:** Broad sense heritability of seven seed and berry-related traits in three grapevine mapping populations

	**MBW**	**MSN**	**TSFW**	**MSFW**	**%SDM**	**RESN**	**RESFW**
MTP3140	0.83	0.73	0.95	0.95	0.94	0.93	0.89
MTP3234	0.73	0.71	0.76	0.75	0.57	0.84	0.80
SxG	0.79	0.74	0.61	0.69	-	0.91	0.91

MBW: mean berry weight; MSN: mean seed number; TSFW: total seed fresh weight; MSFW: mean seed fresh weight; %SDM: seed dry matter percentage; RESN: residual berry weight unexplained by seed number; RESFW: residual berry weight unexplained by total seed fresh weight.

### QTL analysis

**Figure 2:**
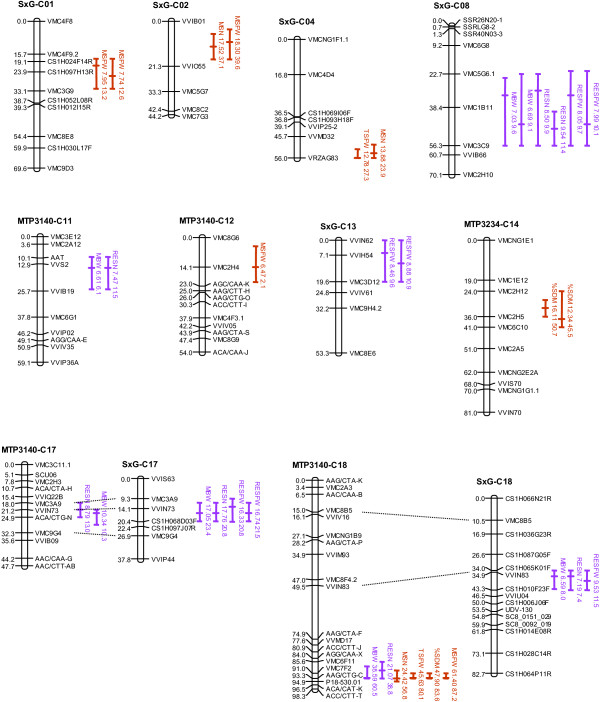
**Stable QTLs for seven seed and berry-related traits in three grapevine mapping populations (consensus maps).** The confidence intervals (CIs) shown are for the inter-year BLUPs of the traits for which a QTL overlapping with this CI was also found in at least two different years. For each CI, the trait abbreviation is followed by the maximum LOD value and the percentage of total variance explained by the QTL. Overlapping confidence intervals for a given trait mean that several LOD peaks were present. Distances are in Kosambi cM. Berry-related traits are in purple and seed-related traits in brown. MBW: mean berry weight; MSN: mean seed number; TSFW: total seed fresh weight; MSFW: mean seed fresh weight; %SDM: seed dry matter percentage; RESN: residual berry weight unexplained by seed number; RESFW: residual berry weight unexplained by total seed fresh weight.

QTLs detected from consensus and parental maps are listed in Tables 3 and 4, Additional file 8: Table S6 and Additional file 9: Table S7 (main features of stable QTLs only), and Additional file 10: Table S8 and Additional file 11: Table S9 (detailed features of all QTLs). Confidence intervals are shown on Figure 2 and Additional file 12: Figure S3 and Additional file 13: Figure S4. In the following, we will focus on the QTLs significant over at least two years, hereafter called stable QTLs: seven for berry weight, four for seed number, six for seed weight, three for seed dry matter.

**Table 3:** Main QTLs for seven seed and berry-related traits in three grapevine mapping populations (consensus maps)

**Trait**^**1**^	**LG**	**Population**	**Years**	**CI extremes**	**Max LOD peak**	**Max % variance**	**Largest allelic effects**^**2**^	**QTL already published (References)**
MBW	8	SxG	05,06	21.2-56.3	8.5	16	Af,D	No
MBW	11	MTP3140	95,98	0-25.7	6.6	11	Af,Am	No
MBW	17	MTP3140	95,98,99	18-28.3	10.3	13	Am,D	No
MBW	17	SxG	05,06,07	9.3-20.4	17.1	31	Am	No
MBW	18	MTP3140	94,95,96,98,99	87.6-93.3	38.6	61	Af,Am	Yes [25,27-29]
MBW	18	SxG	06,07	32.6-43.3	6.6	9	Am,D	No
RESFW	8	SxG	05,06,07	9.2-56.3	10.7	17	Af,Am	No
RESFW	13	SxG	05,06,07	0-32.2	9.7	18	Af,Am	No (but MBW [43])
RESFW	17	SxG	05,06,07	9.3-20.4	16.7	24	Af,Am	No
RESFW	18	SxG	06,07	34-43.3	9.5	12	Am	No
RESN	8	SxG	05,06	22.7-56.3	9.5	15	Af	No
RESN	11	MTP3140	94,95,98	0-33.7	7.5	18	Af,Am	No
RESN	17	MTP3140	94,95,98	18-30.3	8.8	13	Am	No
RESN	17	SxG	05,06,07	9.3-22.4	17.8	29	Am	No
RESN	18	MTP3140	94,95,96,98,99	84-93.3	21.1	45	Af,Am,D	No
RESN	18	SxG	06,07	32.6-43.3	7.2	10	Am	No
MSN	2	SxG	05,06,07	2-23.3	24.8	48	Af,Am,D	Yes [29]
MSN	4	SxG	05,06,07	47.7-56	15.8	29	Af	No
MSN	18	MTP3140	94,98,99	89.6-98.3	24.4	59	Af,Am,D	Yes [25,28]
TSFW	4	SxG	06,07	51.7-56	14.2	29	Af	No
TSFW	18	MTP3140	96,98,99	89.6-93.3	45.6	82	Af,Am	Yes [25,27,28]
MSFW	1	SxG	06,07	14-33.1	8.0	15	Af,Am	No (but TSFW [27])
MSFW	2	SxG	05,06,07	0-33.3	21.4	45	Am,D	No
MSFW	12	MTP3140	95,99	0-23	6.7	3	Am,D	No
MSFW	18	MTP3140	94,95,98,99	87.6-98.3	61.4	87	Af,Am,D	Yes [25,29]
%SDM	14	MTP3234	03,04	24-41	16.1	51	Am,D	No
%SDM	18	MTP3140	98,99	87.6-93.3	47.9	84	Af,Am	Yes [25,29]

QTLs derived with the MQM method. Only the QTLs found for at least two years are presented. All QTLs in this table were also significant for the BLUP of the studied trait. Confidence Interval (CI) extremes were the extremes of all CIs of both year-specific and BLUP QTLs. The genome-wide first type error rate was *α* = 0.05.

^1^MBW: mean berry weight; MSN: mean seed number; TSFW: total seed fresh weight; MSFW: mean seed fresh weight; %SDM: seed dry matter percentage; RESN: residual berry weight unexplained by seed number; RESFW: residual berry weight unexplained by total seed fresh weight.

^2^Major allelic effects: Af female, Am male, D dominance.

**Table 4:** Main QTLs for seven seed and berry-related traits in three grapevine mapping populations (parental maps)

**Trait**^**1**^	**LG**	**Population**	**Map**	**Years**	**CI extremes**	**Max LOD peak**	**Max %var**	**QTL already published (References)**
MBW	8	SG	S	05,06,07^2^	0.0-37.4	9.4	20	No
MBW	13	SG	S	05,06	0.0-13.3	5.9	17	Yes [43]
MBW	17	SG	G	05,06,07	9.9-18.3	15.0	25	No
MBW	18	MTP3140	F	94,95,96,98,99	51.0-58.2	16.3	37	Yes [25,27-29]
MBW	18	MTP3140	M	94,95,96,98,99	101.5-115.3	12.7	32	Yes [25,27-29]
RESFW	8	SG	S	05,06,07	0.0-33.4	8.4	20	No
RESFW	13	SG	S	05,07	0.0-12.0	5.5	14	No (but MBW [43])
RESFW	17	SG	G	05,06,07	9.9-18.3	15.3	24	No
RESFW	17	MTP3140	M	94,95,98,99	17.5-31.2	5.7	15	No
RESFW	18	SG	G	05,06,07	24.2-49.7	5.7	10	No
RESN	1	SG	G	05,06	12.1-41.9	4.9	9	No
RESN	8	SG	S	05,06,07	0.0-39.4	8.7	20	No
RESN	11	MTP3140	F	94,96	10.1-22.9	6.0	20	No
RESN	17	SG	G	05,06,07	8.0-18.3	14.3	23	No
RESN	17	MTP3140	M	94,95,98	17.5-31.2	6.2	16	No
RESN	18	MTP3140	F	95,98,99	51.0-76.9	8.2	22	No
RESN	18	MTP3140	M	94,95,98,99	89.9-115.3	8.7	22	No
MSN	2	SG	G	05,07	4.0-25.9	7.8	20	Yes [29]
MSN	4	SG	S	05,06,07	40.3-48.3	13.6	26	No
MSN	18	MTP3140	F	94,95,96,98	40.8-72.9	11.5	29	Yes [25,28]
MSN	18	MTP3140	M	94,96,98	113.5-115.3	6.5	17	Yes [25,28]
TSFW	4	SG	S	05,06,07	42.3-48.3	16.5	32	No
TSFW	13	SG	G	05,07^2^	13.6-46.4	4.7	11	No (but MSFW [29])
TSFW	18	MTP3140	F	94,95,96,98,99	51.0-58.2	20.3	44	Yes [25,27,28]
TSFW	18	MTP3140	M	94,95,96,98	113.5-115.3	16.5	40	Yes [25,27,28]
MSFW	2	SG	G	05,07	0.0-17.0	9.1	23	No
MSFW	18	MTP3140	F	95,98,99	51.0-56.6	19.3	43	Yes [25,29]
MSFW	18	MTP3140	M	94,95,96,98^2^	101.5-115.3	12.4	35	Yes [25,29]
%SDM	18	MTP3140	F	94,95,96,98,99	46.7-58.2	17.8	41	Yes [25,29]
%SDM	18	MTP3140	M	94,95,96,99^2^	101.5-115.3	13.6	37	Yes [25,29]

QTLs derived with the CIM method. Only the QTLs found for at least two years are presented. All QTLs in this table were also significant for the BLUP of the studied trait, except those indicated by ^2^. Confidence Interval (CI) extremes were the extremes of all CIs of both year-specific and BLUP QTLs. The genome-wide first type error rate was *α* = 0.05.

M: male; F: female; S: Syrah; G: Grenache.

^1^MBW: mean berry weight; MSN: mean seed number; TSFW: total seed fresh weight; MSFW: mean seed fresh weight; %SDM: seed dry matter percentage; RESN: residual berry weight unexplained by seed number; RESFW: residual berry weight unexplained by total seed fresh weight.

^2^QTL not significant for the BLUP of the studied trait.

#### *Berry weight*

Our results on the consensus map confirmed the existence of a major QTL for berry weight already found in seedless populations by several authors on LG 18 near the SSR marker VMC7F2; it was present only in the seedless population MTP3140, explaining 45-61% of total phenotypic variance $\left(\sigma_{P}^{2}\right)$ depending on the year. We also found this QTL for the part of berry weight variation unexplained by seed number (RESN, 24-45%), but not for the part unexplained by total seed weight (RESFW).

In addition to this major QTL, we discovered new, stable QTLs for berry weight on LG 8 (7-16% of $\sigma_{P}^{2}$), LG 11 (10-11%) and LG 17 (7-31%), the latter being present in two populations. A second QTL was found on LG 18, near the VVIN83 marker (9% of $\sigma_{P}^{2}$).

The QTLs for the residual berry weight unexplained by seed number (RESN) were very similar to those for mean berry weight (MBW), whereas important differences were revealed for the residual berry weight unexplained by total seed weight (RESFW). In particular, an additional stable QTL was found for RESFW (8-18%) on LG 13, where none was detected for MBW.

Stable berry weight QTLs showed substantially different patterns between consensus and parental maps. Noticeably, the QTL on LG 13, unstable on the consensus map, was stable for the Syrah parent.

The stable parental QTLs for MBW were found also for RESN and/or RESFW, but four additional stable parental QTLs were found for residual values, on LGs 1, 11, 17 and 18. Among them, the QTL for RESN on LG 1 (6-9%) revealed a new locus involved in berry weight variation that was found neither in consensus maps nor with MBW or RESFW.

No stable QTL for raw or residual berry weight data was found on consensus or parental maps in population MTP3346.

#### *Seed traits*

For mean seed number per berry (MSN), mean seed fresh weight per berry (MSFW), total seed fresh weight per berry (TSFW) and seed dry matter percentage (%SDM), we confirmed the already described major QTL on LG 18 near VMC7F2, that explained up to 59%, 87%, 82% and 35% of the total variance, respectively.

For MSN, we confirmed a QTL previously found on LG 2 (12-48%) and discovered two additional stable QTLs, on LG 4 (14-29%) and LG 14 (17-23%, in MTP3346). For MSFW, three new stable QTLs were discovered on consensus maps on LG 1 (9-15%), LG 2 (13-45%) and LG 12 (3%). For TSFW, two new QTLs were detected, on LG 4 (22-29%, both consensus and parental) and LG 13 (9-11%, parental only). For %SDM, two other QTLs were uncovered, on LG 5 (17-31%, in MTP3346) and LG 14 (35-46%).

### Candidate genes

Despite having reduced explored intervals to a distance of +/- 3 cM around the LOD peak, the number of positional candidate genes found remained very large, varying from 77 to 167 across QTL regions (Additional file 14: Table S10). However, only 11 corresponded to functional candidate genes involved in cell wall modifications, water transport, transcription regulation, organ identity, ethylene and auxin signalling in grapevine. Three additional ones belonged to the gene families with an established role in fruit weight in tomato (Table 5 and Additional file 15: Table S11). Indeed, the recent cloning of the fruit weight QTL *fw3.2* in *Solanum lycopersicum* (cultivated tomato) [58] led to the identification of the LOC101258933 gene, encoding a cytochrome P450 78A-like protein. In the grapevine genome, eight genes are annotated as P450 78A-like proteins (NCBI). Noticeably, the putative ortholog of LOC101258933, displaying 75% identity on 99% of the sequence length (LOC100253660, LG17:5600143..5602650), colocalized with the major berry weight QTL, while the second matching gene did not colocalize with a QTL (LOC100249034, LG1:381267..382921, 71% identity on 99% of the sequence).

**Table 5:** Published functional candidate genes potentially involved in grapevine seed and/or berry development colocalized with QTLs detected

**Grapevine 12X gene ID**	**Gene name**	**LG**	**position in 12X**	**putative homologous gene and/or function**	**In the CI of a QTL of the present study**^**1**^	**Reference**
GSVIVT01011687001	*-*	1	5123090 5124512	*ZmCNR2* involved in tissue growth activity in maize	RESN, MSFW	[60]
GSVIVT01018839001	VvAP3.2 = *TM6*	4	19395438 19397804	*MIKC* gene expressed in flowers and berries	MSN, TSFW	[61]
GSVIVT01025701001	-	8	12843442 12845830	ethylene signalling protein preferentially expressed in flb mutant vs WT	MBW - RESFW	[62]
GSVIVT01025700001			12845836 12854444			
-	*-*	8	13327417	cell number regulator 8-like	MBW, RESN, RESFW	-
			13332631			
GSVIVT01032681001	*EXP2*	13	1578645 1580497	expansin *EXP8* (*A. thaliana*) with expression linked to berry development	MBW, RESFW (in Syrah)	[63]
GSVIVT01016525001	*EXP3*	13	3120277 3122170	expansin *EXP4* (*A. thaliana*) with expression linked to berry development	MBW, RESFW (in Syrah)	[63]
GSVIVT01016276001	AQ2 = *PIP2;1*	13	5602025 5605019	Plasma membrane intrinsic protein 2B (PIP2B); aquaporin *PIP2.2* (*A. thaliana*) with expression linked to berry development	RESFW	[63]
GSVIVT01008122001	-	17	5600143 5602650	Cytochrome P450 78A-like protein in *Solanum lycopersicum* (tomato), harboring the causal SNP of a berry weight QTL	MBW-RESN-RESFW	[58]
GSVIVT01008046001	-	17	6316168 6320317	*WRKY* transcription factor 72-like, regulation of skin and flesh ripening	MBW, RESN, RESFW	[64]
GSVIVT01008034001	-	17	6455525 6456835	transcription factor *bHLH135*-like, regulation of pre-*véraison* processes in the pericarp	MBW, RESN, RESFW	[64]
GSVIVT01007987001	*EXPA*	17	6888373 6890086	Alpha-expansin with expression linked to berry development	MBW^2^, RESN^2^, RESFW^2^	[65]
GSVIVT01009791001	*VvBG1*	18	11286578 11293205	beta-galactosidase (cell-wall modifying enzyme expressed during berry development)	MBW, RESN, RESFW	[66]
GSVIVG01009815001	*VvAP3*	18	11506514 11512366	*Apetala 3* (Arabidopsis), MADS box flower development	MBW, RESN, RESFW	[67]
				expressed highest in young fruit		[68]
				expressed almost exclusively in inflorescences		[61]
GSVIVT01009865001	*-*	18	11920498 11929437	auxin response factor 5-like, transcript variant 1, includes an EST of hypothetical transcription factor preferentially expressed in WT vs flb mutant	RESFW	[62]
GSVIVG01025945001	*VvAGL11 = VvAG3 = MADS5*	18	26888677 26896544	*Agamous like 11* (Arabidopsis), MADS box ovule identity (*MIKC* gene expressed in flowers and berries)	MBW, RESN, MSN, TSFW, MSFW,%SDM	[30,67]

Candidate genes colocalized with QTLs detected in the present study. Gene numbers were obtained from the automatic annotation provided with the Genoscope 12X whole genome sequence release of PN40024 (http://www.genoscope.cns.fr/externe/GenomeBrowser/Vitis/[1]).

^1^MBW: mean berry weight; MSN: mean seed number; TSFW: total seed fresh weight; MSFW: mean seed fresh weight; %SDM: seed dry matter percentage; RESN: residual berry weight unexplained by seed number; RESFW: residual berry weight unexplained by total seed fresh weight.

^2^In QTL confidence interval but not within 3 cM of LOD peak position.

The cloning of another fruit weight QTL in tomato, *fw2.2 *[69], led to the identification of the LOC101245309 gene, encoding a cell number regulator. The three best matches on *Vitis* refseq database were on LG3, where no QTL was found. However, three other genes were annotated as predicted cell number regulator-like in grapevine genome, among which LOC100246592 colocalized with a berry size QTL, on LG8 (Additional file 16: Table S12). In addition, the best match of the maize *ZmCNR2* gene from the same family, potentially involved in tissue growth [60], was obtained for LOC100250939, encoding a plant cadmium resistance 2-like protein, which colocalized with the parental-only berry growth QTL on LG1.

Many other positional candidate genes with putative functions similar to the functions of these 14 genes were found under berry weight QTLs, but no published study provided evidence of any function related to grapevine berry development (Additional file 16: Table S12).

## Discussion

### Genetic architecture of berry weight and seed content

### Detection of new QTLs

This study reports five new QTLs for berry weight in grapevine, located on LGs 1, 8, 11, 17 and 18, and stable over at least two years. The only stable QTLs previously known for this trait were a major one linked to seedlessness on LG 18 [25,27-29] and two QTLs on LGs 5 and 13 found in a complex hybrid cross including non-*vinifera* background [43]. The second QTL for berry weight found on LG 18, near the VVIN83 marker, was stable over two years in our study, whereas it was found unstable by Cabezas et al. [27]. All these novel QTLs were stable over time but not over populations, except the QTL with the largest effect on LG 17, found in two populations with distant genetic backgrounds. This is consistent with the highly composite nature of berry weight, affected by numerous factors (cell multiplication, cell wall modifications, photosynthesis, sucrose and water transport, growth regulators, etc.), and therefore expected to be under polygenic control, with different causal polymorphisms segregating in different populations.

The present work is the first report of a stable QTL for seed traits in a seeded wine grape background, on LG 4. New QTLs for seed traits in seedless and/or table backgrounds were also found, on LGs 5, 12 and 14.

A number of methodological choices have allowed us to make improvements over previous studies and therefore to find additional QTLs and to provide new insights for already known ones. First, the simultaneous study of four populations using shared methods for phenotyping, genotyping and statistical analyses facilitated result comparisons. In previous studies (Additional file 1: Table S1), comparisons were made difficult by differences in thresholds (genome-or chromosome-wide), maps (parental or consensus) and studied traits, even if most studies used a common phenotyping protocol (European project MASTER, ended in 2005). Several QTLs could not have been found using parental maps only as in [25] (Tables 3 and 4). Most of these showed dominance allelic effects on the consensus map. However, the study of parental maps proved to remain necessary by revealing QTLs otherwise unstable on the consensus map. This could result from a higher power of additive QTL detection in parental maps, where the sample size of each genotypic class is twice as large as in the consensus map. Second, composite interval mapping allowed to discover additional QTLs, compared to interval mapping only as in [28], such as the berry weight QTL on LG 17, detected after adding the major QTL on LG18 as a cofactor in population MTP3140. Third, by analysing mean seed fresh weight in addition to total seed fresh weight, three additional QTLs could be discovered. Moreover, the absence of QTL for mean seed weight on LG 4 suggests that the only QTL for total seed weight on this LG was most probably due to the QTL for seed number. This clearly emphasizes the need to study elementary components of complex traits in QTL detection. Fourth, searching for QTLs using BLUPs of the traits of interest in addition to QTLs in individual years provides useful summarized information. Despite its demonstrated interest in other plant species [70], the use of BLUPs in QTL studies on grapevine is very recent [49,71,72] and was applied here to berry weight or seed traits for the first time. Last, we provide here the first attempt to search for QTLs using the residual part of berry weight unexplained by seed trait variation. This analysis successfully revealed two stable QTLs for residual berry weight on LGs 1 and 13, that were not detected with berry weight raw data. The QTL found on LG 1 had never been reported before.

#### *Confirmed or invalidated QTLs*

We found only in the seedless population MTP3140 the major QTL on LG 18 for berry weight and seed traits previously found in several studies [25,27-29]. In the second seedless population MTP3346, the parents were homozygous around this QTL, therefore no segregation occurred. In this region, no stable QTL was found for the residual part of berry weight variation not explained by total seed weight, whereas a stable QTL was found for the part unexplained by seed number alone. This difference could be due to insufficient power to detect QTLs with a low residual variation since the genetic correlation between berry weight and total seed weight was very high. Alternatively, it could suggest that this major berry weight QTL is a pleiotropic QTL for both mean seed fresh weight and berry weight, with direct or indirect effects via growth regulator production by seeds, as argued by Meijia et al. [30].

The stable QTLs found here were consistent with several previously reported QTLs for related traits, and their comparison provided more insight into their determinism. On LG 1, Cabezas et al. [27] found a stable QTL for total seed fresh weight that overlapped the QTL for mean seed fresh weight in our study, suggesting that the underlying gene might control total seed weight through individual seed weight (not studied by these authors) rather than through seed number. On LG 13, our QTL for berry weight colocalized with the stable one found by Fisher et al. [43] in a complex interspecific background. In the same region, we also found a QTL for the residual berry weight unexplained by total seed fresh weight, which suggests that this berry weight QTL was not only due to variation in seed content. On LG 13, we detected a QTL for total seed fresh weight where Costantini et al. [29] found a QTL for mean seed fresh weight, which is consistent since these authors did not study total seed fresh weight, and which suggests that our QTL for total seed fresh weight is explained by mean seed weight rather than by seed number.

On LG 2, the QTLs detected for seed number and mean seed fresh weight colocalized with the QTL for seed number in Costantini et al. [29]. However, in a further analysis to include flower sex morphology (females *vs* hermaphrodites) as a covariate into the QTL detection model using the R/qtl package [73], our large seed QTLs on LG 2 were not detected (data not shown). This suggests these QTLs were not true QTLs for seed traits but corresponded to the major locus for sex mapped on this LG by several authors [74-77]. The SxG population segregated for sex since both parents were heterozygous HF (hermaphrodite-female). The effect of sex on both seed number and mean seed fresh weight was highly significant each year (*P* < 0.001). The QTL for seed number reported by Costantini et al. [29] on LG 2 is most probably also explained by the sex locus which segregated in their mapping population (L. Costantini, pers. comm.). To our knowledge, there is no published evidence of an effect of sex on seed number or weight in grapevine, even though seed number in female plants is expected to be lower than in hermaphrodite ones as suggested by the obligatory outcrossing and higher rate of parthenocarpic berries set [78]. Interestingly, though female varieties usually have rather large berries [3], we found no QTL for berry weight on LG 2 and the effect of sex on berry weight was never significant in the SxG population for this trait.

Only two stable QTLs for berry weight previously published, on LGs 5 and 10, were not found here. This highlights the large genetic variability explored in our study by involving eight different parents. A total of at least six stable minor QTLs for seed traits in seedless background were found in this study and/or in previous studies (Additional file 1: Table S1). Therefore, the genetic model for seedlessness proposed by Bouquet and Danglot [46], with a major gene on LG 18 regulating three minor genes, needs to be complexified.

Only one previously reported unstable QTL [27] was found stable in one of our populations (on LG 18 near VVIN83). All other published unstable QTLs (Additional file 1: Table S1) were not confirmed in our study, emphasizing the low potential interest of such QTLs interacting with environment. Therefore, we chose not to discuss unstable QTLs, even those concerning regions not identified previously.

#### *Heritability*

The relatively high values of broad sense heritabilities found for berry and seed weights in the four populations (0.51-0.93 and 0.61-0.95, respectively) were consistent with previously published results [39-42]. However, heritability for seed number was much higher in our study than in Daulta et al. [39] (≥ 0.59 vs 0.34). The allelic effects of all QTLs found here had at least one additive component, and about two thirds of the QTLs did not show any large dominance effect (Table 3 and Additional file 8: Table S6). This is consistent with the high values of narrow sense heritabilities (around 0.60) reported for both berry weight and seedlessness [31,32,38].

### Relationship between berry weight and seed traits

Most stable QTLs found here for berry weight and seed traits did not colocalize. In particular, the seed QTL on LG 5 did not colocalize with the berry weight QTL found by Fisher et al. [43]. Moreover, it is worth noting that no colocalization was found with the two minor QTLs for seed number. Nevertheless, colocalization was observed in three regions. The first case concerned the major QTL on LG 18 in the MTP3140 seedless population. This is consistent with the high genetic correlation observed in this population and the weaker correlation obtained for the other populations with no QTL found in this part of LG 18. This result is also consistent with the strong correlations previously reported in segregating populations or collections involving seedless genotypes [24-32], mainly derived from cv. Sultanina, and the absence of correlation reported in collections composed mainly of seeded cultivars [4,33]. In the seeded population SxG, the extent of variation in seed traits was smaller compared to MTP3140. However, this alone could not explain the lower berry-seed correlation, since variation was large in MTP3234. The second case of berry-seed QTL colocalization was on LG 1. But the consensus QTL for mean seed weight had both cvs. Syrah and Grenache additive effects, whereas the QTL for residual berry weight was stable only in Grenache. This indicates that colocalization could not result only from a pleiotropic effect. The third colocalization was found on LG 13 with a QTL for total seed weight in cv. Grenache. In that case also, pleiotropy alone could not explain colocalization because additive effects had different parental origins.

From an evolutionary point of view, the wild *V. vinifera* subsp. *sylvestris* has much smaller berries than the cultivated *V. vinifera* subsp. *sativa* but seeds of similar size [79,80]. Therefore, the anthropic selection for larger berry sizes was probably based on QTLs for berry size unrelated to seed traits, such as the four ones detected in this study. Quite interestingly, the QTL on LG 17 colocalized with the 5-Mb candidate domestication locus on chromosome 17 found by Myles et al. [81]. If selection occurred during primary domestication, a drastic reduction in genetic diversity would be expected at this locus in cultivated grapevine. However, small berry size alleles may have been re-introduced afterwards during secondary phases of domestication [82,83], or mutations may have arisen after domestication, which could explain the detection of this QTL in two different populations. Alternatively, it cannot be excluded that these two populations harbor rare alleles.

In the future, these limited colocalizations between QTLs for berry size and seed traits offer the possibility to reduce seed number and size without reducing berry size using Marker Assisted Selection (MAS) to accelerate table grape breeding. However, the existence of a threshold in seed content, under which the potential berry size would be limited, could prevent the complete dissociation of these traits and therefore the breeding of cultivars with very large berries and seeds imperceptible enough for the consumers.

The role in grape berry development of growth regulators produced by seeds was questioned by Mullins et al. [6]. It is generally accepted that embryos control cell division in the surrounding fruit tissues [84]. In grape, Mullins et al. [6] argued that growth regulators produced by seeds might not play a major role in berry development pattern in grapevine, since the relationship between mean growth patterns of seeds and berries is highly variable among seeded cultivars. Sugar accumulation or cell wall extensibility could better explain grape berry growth pattern. However, Ojeda et al. [7] suggested that seed growth affects more profoundly berry cell mitosis than cell enlargement. The distinct QTLs found for seed and berry traits in our study strengthen Mullins et al.’s hypothesis [6], since berry QTL intervals harbored several genes involved in transport or cell wall modifications (see below). Alternatively, QTLs for berry size that did not colocalize with QTLs for seed content might be QTLs for some other internal factors related to berry size at maturity, such as growth regulators not produced by seeds or sink/source *ratio *[9]. Measurements of these traits on the same populations would be required to assess the extent of their genetic relationship with berry weight at maturity.

### Candidate genes

Several hundred positional candidate genes were found under QTLs (Additional file 14: Table S10). However, only a few genes deserve particular attention (Table 5), because they could be either potential orthologs of genes underlying tomato fruit weight QTLs, or involved in berry weight or seed content based on functional evidence in grapevine.

A cytochrome P450 78A gene partly controlling fruit weight in tomato was suspected to be involved in the domestication of this species [58]. The colocalization between the putative ortholog of this gene and the large berry size QTL on LG17 suggests a rather strict conservation of the control of fruit size across such physiologically distant fruit species. Selection of large-fruit alleles at this locus during the domestication process is also possible in grapevine since it colocalizes with a putative domestication locus [81]. Colocalization was also observed between the berry weight QTL on LG8 and a gene from the CNR family, which regulates cell number and was involved in fruit size changes during the domestication of tomato [69]. De Franceschi et al. [57] also suggested the implication of a gene from this family in the domestication of sweet cherry.

Other functions, involved in cell expansion during berry development are likely to be responsible for grapevine berry weight variation, in the first place concerning the increase in cell wall area. Schlosser et al. [63] showed that a cohort of candidate cell wall-modifying enzymes (expansin, glycosyl hydrolase, pectinesterase, pectate lyase, cellulase, XET) were highly expressed during berry growth. The two expansin isogenes that these authors studied, *EXP2* and *EXP3*, fell under the berry weight QTL on LG 13. An alpha-expansin with expression linked to berry development has also been found in the confidence interval of the berry weight QTL on LG 17 [65]. On LG 18, a beta-galactosidase gene expressed during berry development colocalized with the minor berry weight QTL [66]. This enzyme showed the most dramatic change in activity amongst cell wall enzymes during berry ripening, probably linked to the increase in pectin solubility [85].

Water import, mediated by *MIP* and *PIP* genes, is also essential for cell expansion. On LG 13, the putative aquaporine *VvPIP2;1* isogene, up-regulated at the beginning of the second growth phase [63], was located under the QTL for residual berry weight. An EST-derived probe of PIP2-1 type was found over-expressed after GA-treatment around *véraison *[86]. A putative paralog with similar function, Refseq *VvPIP2;3*, represents the best hit of EF364437.1 in the 12X genome and is located under the QTL for berry weight on LG 8. *VvPIP2;1* and *VvPIP2;3* actually triggered large water channel activities upon expression in *Xenopus* oocytes [87]. These genes were highly expressed in expanding green berries, according to EST counts at NCBI, as confirmed by RNAseq [88].

Many other genes could also be considered as potential candidate genes for berry weight QTLs, even when no precise function has been identified for them directly in grapevine yet (Additional file 16: Table S12). These genes are those expected to control processes involved in fruit expansion, such as auxin and gibberellin signalling [16], source (leaf area) to sink (fruit weight) *ratio *[89], sugar and water balance [9], sugar biosynthesis and transport [65,90], scion/rootstock interaction, cell division or elongation, transcription control [64], signal transduction [90], or organ identity. In particular, the gene coding for an *ERD6*-like putative sugar transporter (GSVIVT01009719001), could be relevant under the berry weight QTL on LG 18 near the VVIN83 SSR. Lastly, it should be noted that *VvGAI1*, a negative regulator of gibberellin response, although present in the confidence interval of the QTL on LG 1, was not expressed in berries and berry weight was not impaired in the *vvgai1* loss-of-function mutant [91].

## Conclusions

In four grapevine seeded and seedless mapping populations, we identified nine new QTLs for berry weight and seed content, which were stable over time. With this single study, we thus increased more than twofold the number of stable QTLs for these traits known to date. Most berry and seed QTLs did not colocalize, providing new insight into the complex correlation between berry size and seed content. Such uncolocalized QTLs could be used in marker-assisted breeding. A few candidate genes under these QTLs were functionally relevant and could readily be further tested by association genetics. However, more than one out of four genes under the QTLs had unknown functions (data not shown). Since over 17,000 genes are known to be expressed during berry development, of which nearly one third are stage-specific [88], expression patterns alone will not be very helpful for further candidate gene screening. For complex traits such as berry weight and seed content at maturity, fine mapping of QTLs and additional functional data are required to discover their causal polymorphisms.

## Abbreviations

BIC: Bayesian Information Criterion; BLUP: Best Linear Unbiased Predictor; CI: Confidence interval; CIM: Composite interval mapping; CTIFL: Centre Technique Interprofessionnel des Fruits et Légumes; IM: Interval mapping; INRA: Institut National de la Recherche Agronomique; KW: Kruskal-Wallis; LG: Linkage group; MAS: Marker assisted selection; MBW: Mean berry weight; MQM: Multiple QTL Model; MSFW: Mean seed fresh weight; MSN: Mean seed number; NCBI: National Center of Biotechnology Information; QTL: Quantitative trait locus; RESFW: Residual berry weight unexplained by total seed fresh weight; RESN: Residual berry weight unexplained by seed number; MAS: Marker assisted selection; %SDM: Seed dry matter percentage; SSR: Simple sequence repeat; TSFW: Total seed fresh weight.

## Competing interests

The authors declare that they have no competing interests.

## Authors’ contributions

AD conceived and designed the study, contributed to the phenotyping of the populations, managed the cultivation and phenotyping, performed statistical and bioinformatical analyses and drafted the manuscript. YB, MG and GB were in charge of the cultivation of the populations in the field and greenhouse. YB, MF, MG, GB, FE, SD, PF, TP, PO, CaR, VL, RB and JPP participated in the phenotyping of the populations. ChR helped with bioinformatic analyses and discussion on candidate genes. CH helped searching for functional candidate genes. VL, RB, ChR, JPP and PT participated in the discussion of results and helped to draft the manuscript. PT coordinated the study and participated in phenotyping. All authors read and approved the final manuscript.

## Supplementary Material

Additional file 1: Table S1 Published QTLs for berry weight and seed traits in *Vitis.* Click here for file

Additional file 2: Table S2 Main features of the framework genetic maps used for QTL detection in four grapevine mapping populations. Click here for file

Additional file 3: Figure S1 Raw phenotypic data distributions for five seed and berry-related traits in four grapevine mapping populations. Click here for file

Additional file 4: Table S3 Transformations applied to raw data and model selected to estimate BLUPs of genetic values and broad-sense heritability, for seven seed and berry-related traits in four grapevine mapping populations. Click here for file

Additional file 5: Table S4 Phenotypic correlations between years for seven seed and berry-related traits in four grapevine mapping populations (Spearman correlation coefficient). Click here for file

Additional file 6: Figure S2 Genetic correlations (among BLUPs of the genetic value) between seven seed and berry-related traits within years in the grapevine mapping population MTP3346. Click here for file

Additional file 7: Table S5 Broad sense heritability of seven seed and berry-related traits in the grapevine mapping population MTP3346. Click here for file

Additional file 8: Table S6 Summary of main QTLs for seven seed and berry-related traits in the grapevine mapping population MTP3346 (consensus map), derived with the MQM method. Click here for file

Additional file 9: Table S7 Summary of main QTLs for seven seed and berry-related traits in the grapevine mapping population MTP3346 (parental maps), derived with the CIM method. Click here for file

Additional file 10: Table S8 Detailed features of all QTLs for seven seed and berry-related traits in four grapevine mapping populations (consensus maps), derived with the MQM method. Click here for file

Additional file 11: Table S9 Detailed features of all QTLs for seven seed and berry-related traits in four grapevine mapping populations (parental maps), derived with the CIM method. Click here for file

Additional file 12: Figure S3 Stable QTLs for seven seed and berry-related traits in the grapevine mapping population MTP3346 (consensus map). Click here for file

Additional file 13: Figure S4 Stable QTLs for seven seed and berry-related traits in four grapevine mapping populations (parental maps). Click here for file

Additional file 14: Table S10 Intervals explored and number of positional candidate genes found. Click here for file

Additional file 15: Table S11 Published functional candidate genes potentially involved in seed and/or berry development not colocalized with QTLs detected in the present study [92-106]. Click here for file

Additional file 16: Table S12 Functional candidate genes comprised within +/- 3 cM of the LOD peaks of consensus berry size QTLs (BLUPs only). Click here for file
