# Supplementary material for: New stable QTLs for berry weight do not colocalize with QTLs for seed traits in cultivated grapevine (Vitis vinifera L.)
Source: BMC Plant Biol. 2013 Dec 19;13:217. doi: 10.1186/1471-2229-13-217 (PMC3878267; doi:10.1186/1471-2229-13-217)
Supplement: Additional file 1: Table S1 — Published QTLs for berry weight and seed traits in Vitis. [file 1471-2229-13-217-S1.pdf]

Additional file 1: Table S1 - Published QTLs for berry weight and seed traits in *Vitis*

Trait	LG	Doligez et al., 2002	Fischer et al., 2004[1]	Cabezas et al., 2006	Mejia et al., 2007	Costantini et al., 2008[2]
MBW	1					18
	5		**?**[4]			
	12					9
	13		**?**[4]			
	15			19		
	18-7F2	**38**[2]		**44**	**67**	**43**
	18-N83			23		
MSN	2		nr			**23**
	8	11				
	11			67		
	18-7F2	**48**[3]			**48**	
TSFW	1		nr	**15**		nr
	10			**33**		
	18-7F2	**49**[3]		**63**	**84**	
MSFW	6		nr	nr	nr	21
	10					13
	13					**16**[5]
	15					13
	18-7F2	**38**[3]				**28**
%SDM	18-7F2	**40**[3]	nr		64	**91**

QTLs detected with a genome-wide first-type error rate of 5%. The reported number is the maximum % of total variance explained among years and maps (parental and consensus), in bold when the QTL was present in at least 2 years. nr: trait not reported.
The results of Fanizza et al. (2005) were not included because they were based on a subset of the data reported in Costantini et al. (2008); those of Mejia et al. (2011) were not included because the QTLs on LGs others than LG 18 were not reported.
MBW: mean berry weight; MSN: mean seed number; TSFW: total seed fresh weight; MSFW: mean seed fresh weight; %SDM: seed dry matter percentage; RESN: residual berry weight unexplained by seed number; RESFW: residual berry weight unexplained by total seed fresh weight

[1] Complex hybrid cross including up to seven American *Vitis* species
[2] LOD threshold was extrapolated from the available chromosome-wide values to 3.5 for parental maps and 4.5 for consensus map, to reflect usual values of genome-wide values
[3] LG determined according to Doligez et al., 2010
[4] Stable QTL detected but magnitude of effect not reported
[5] Doubtful QTL (in a large gap of 26.4 cM, with no strong significance of KW test)
