# Supplementary material for: New stable QTLs for berry weight do not colocalize with QTLs for seed traits in cultivated grapevine (Vitis vinifera L.)
Source: BMC Plant Biol. 2013 Dec 19;13:217. doi: 10.1186/1471-2229-13-217 (PMC3878267; doi:10.1186/1471-2229-13-217)
Supplement: Additional file 2: Table S2 — Main features of the framework genetic maps used for QTL detection in four grapevine mapping populations. [file 1471-2229-13-217-S2.pdf]

Additional file 2: Table S2 - Main features of the framework genetic maps used for QTL detection in four grapevine mapping populations.

Progeny	MTP3140			MTP3234			SxG			MTP3346[1]		
Map	F	C	M	F	C	M	S	C	G	F	C	M
Total # of loci	187	233	199	96	119	105	121	153	133	25	29	26
Total length (cM)	1079	1204	1143	935	1267	1365	986	1116	1170	260	290	235
Mean inter-marker distance (cM)	6.4	5.6	6.4	12.1	12.7	15.9	9.7	8.3	10.3	17.3	15.3	14.7
# gaps > 20 cM	3	5	5	12	17	25	6	10	17	4	5	4
# gaps > 30 cM	0	1	2	3	3	8	0	0	3	1	0	0

[1]partial map in only ten genomic regions of 15.9-48.9 cM

Maps were already published for MTP3140 [87], MTP3234 [47] and MTP3346 [87]

F female, C consensus, M male, S Syrah, G Grenache, #: number of; cM: centiMorgan
