# Supplementary material for: New stable QTLs for berry weight do not colocalize with QTLs for seed traits in cultivated grapevine (Vitis vinifera L.)
Source: BMC Plant Biol. 2013 Dec 19;13:217. doi: 10.1186/1471-2229-13-217 (PMC3878267; doi:10.1186/1471-2229-13-217)

Additional file 3: Figure S1 - Raw phenotypic data distributions for five seed and berry-related traits in four grapevine mapping populations. A. MBW: mean berry weight (g), B. MSN: mean seed number, C. TSFW: total seed fresh weight (mg), D. MSFW: mean seed fresh weight (mg), E. %SDM: seed dry matter percentage. Mean parental values: triangle for MTP2223-27 and Syrah; cross for MTP2121-30 and Grenache. Parental values were not available for MTP3140 in 1994 and 1996, MTP3234 and MTP3346.

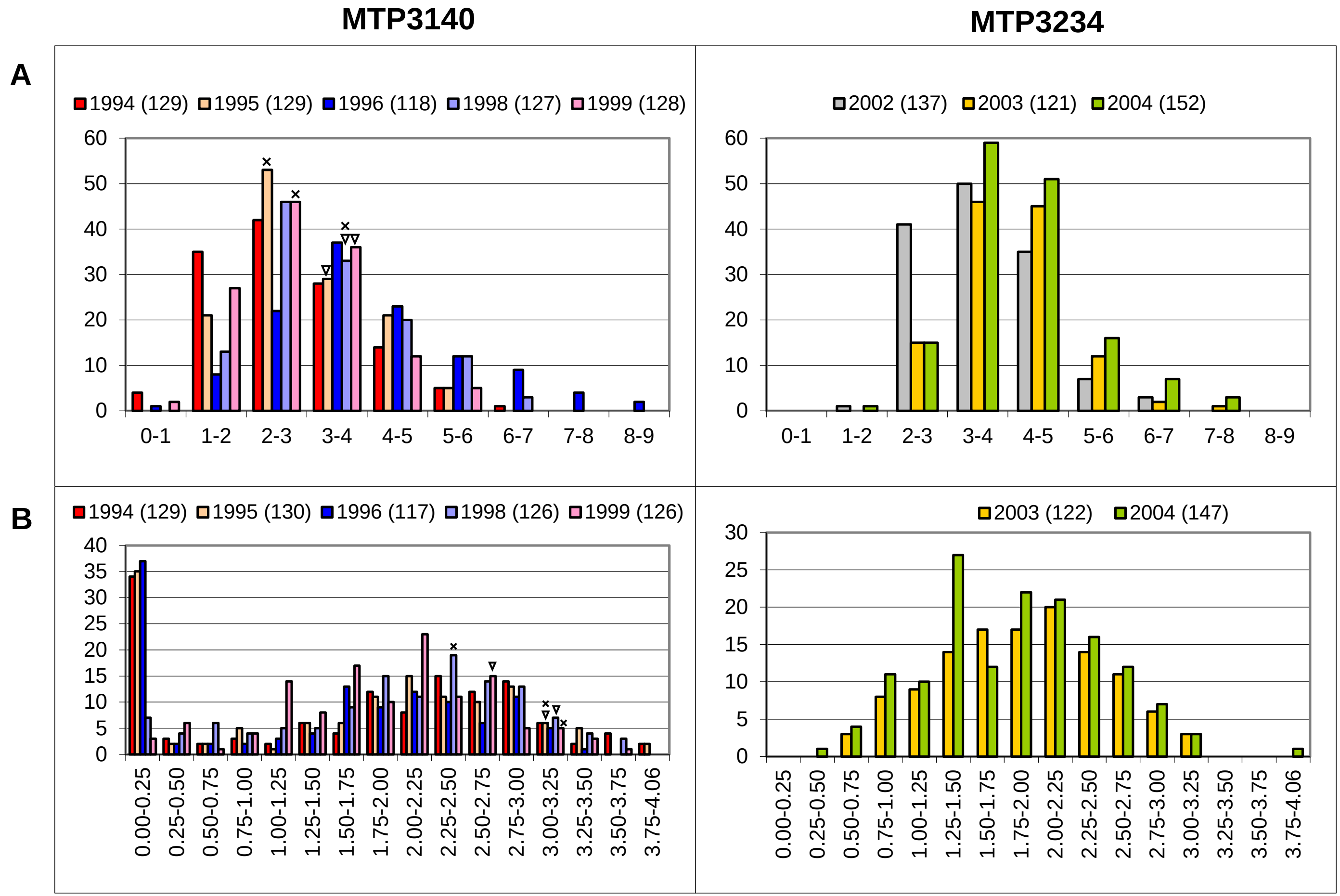

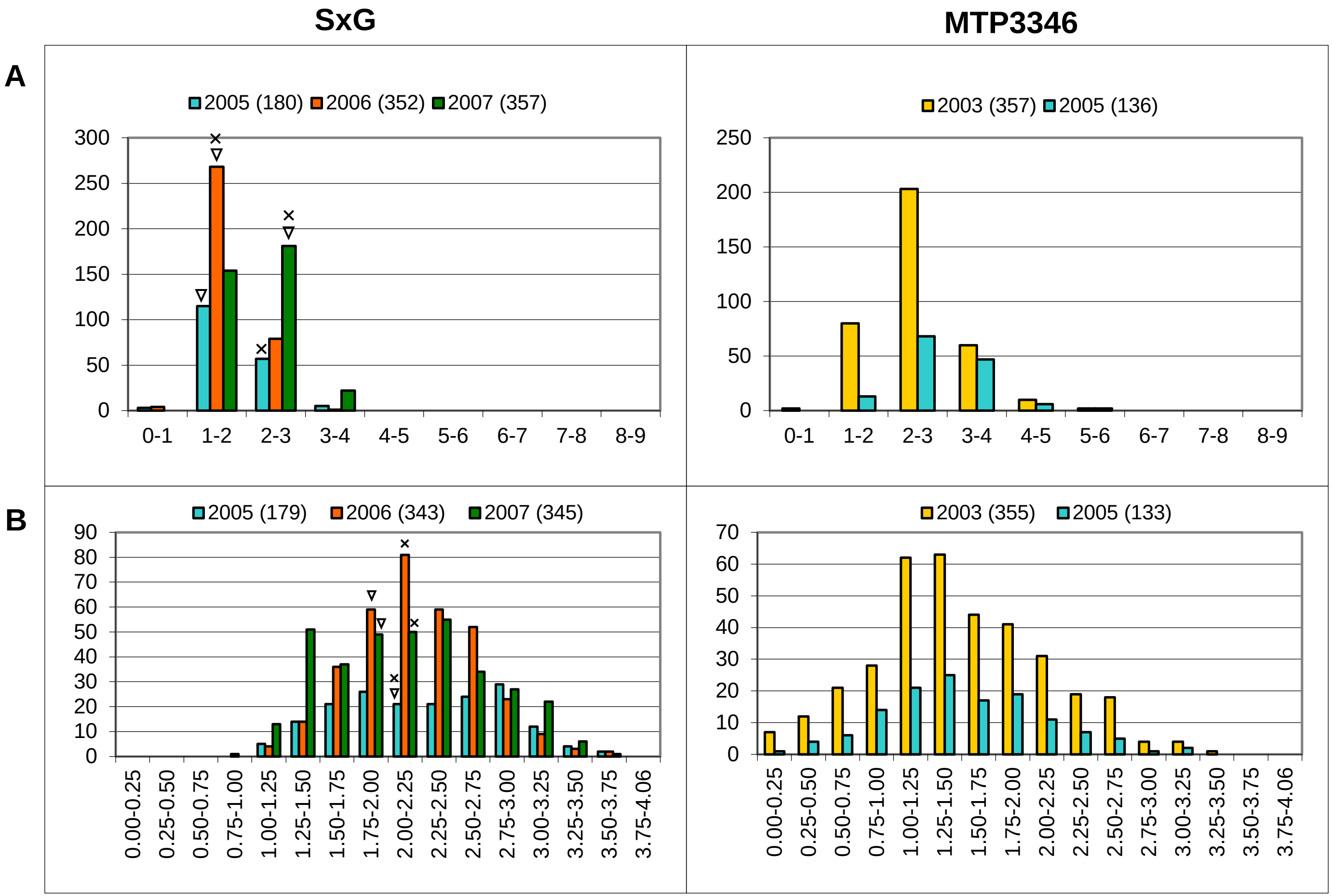
SxG
MTP3346
A
2005 (180) 2006 (352) 2007 (357)
2003 (357) 2005 (136)
0-1
1-2
2-3
3-4
4-5
5-6
6-7
7-8
8-9
B
2005 (179) 2006 (343) 2007 (345)
2003 (355) 2005 (133)
0.00-0.25
0.25-0.50
0.50-0.75
0.75-1.00
1.00-1.25
1.25-1.50
1.50-1.75
1.75-2.00
2.00-2.25
2.25-2.50
2.50-2.75
2.75-3.00
3.00-3.25
3.25-3.50
3.50-3.75
3.75-4.06

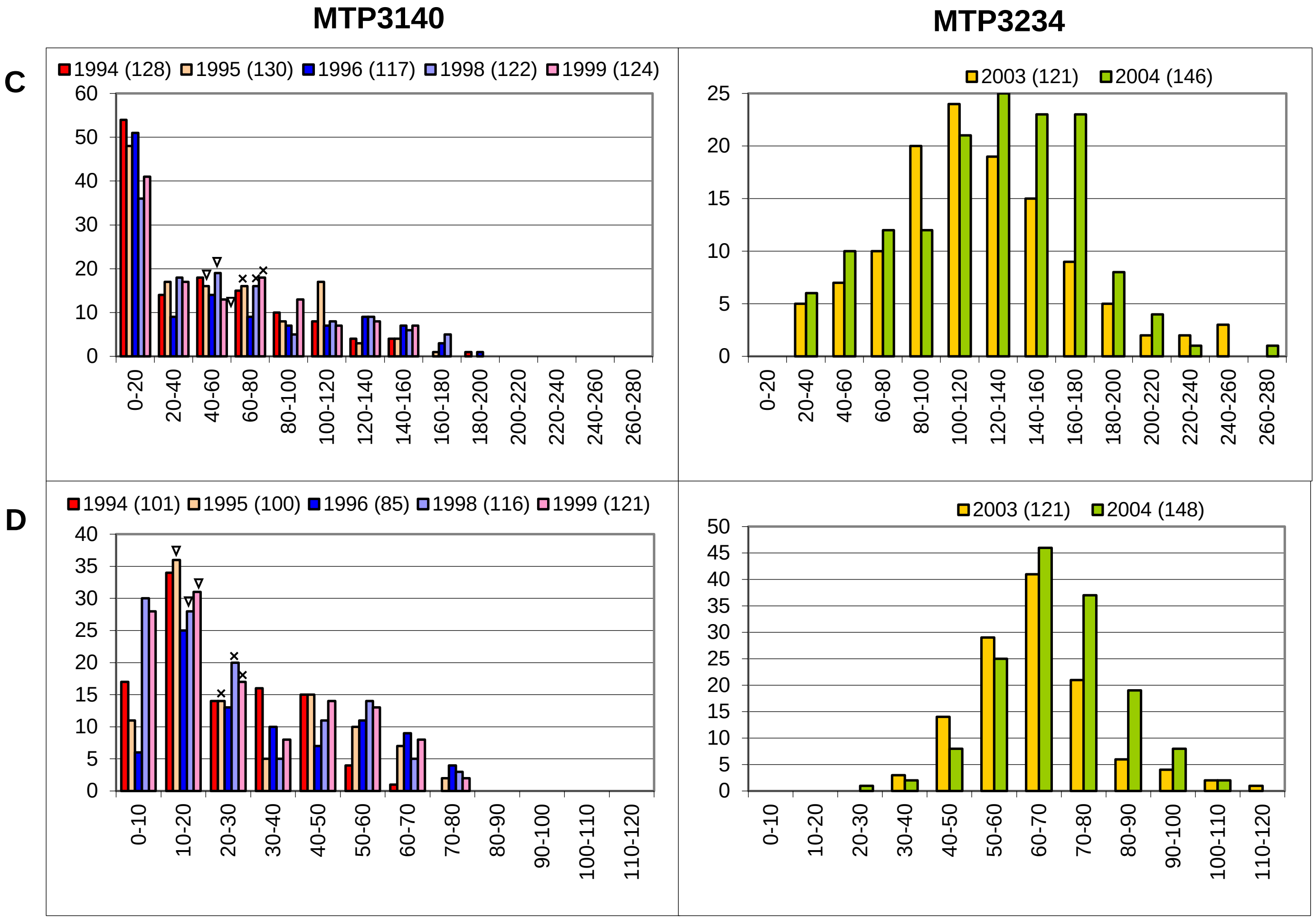
MTP3140
MTP3234
C
1994 (128)
1995 (130)
1996 (117)
1998 (122)
1999 (124)
2003 (121)
2004 (146)
0-20
20-40
40-60
60-80
80-100
100-120
120-140
140-160
160-180
180-200
200-220
220-240
240-260
260-280
D
1994 (101)
1995 (100)
1996 (85)
1998 (116)
1999 (121)
2003 (121)
2004 (148)
0-10
10-20
20-30
30-40
40-50
50-60
60-70
70-80
80-90
90-100
100-110
110-120

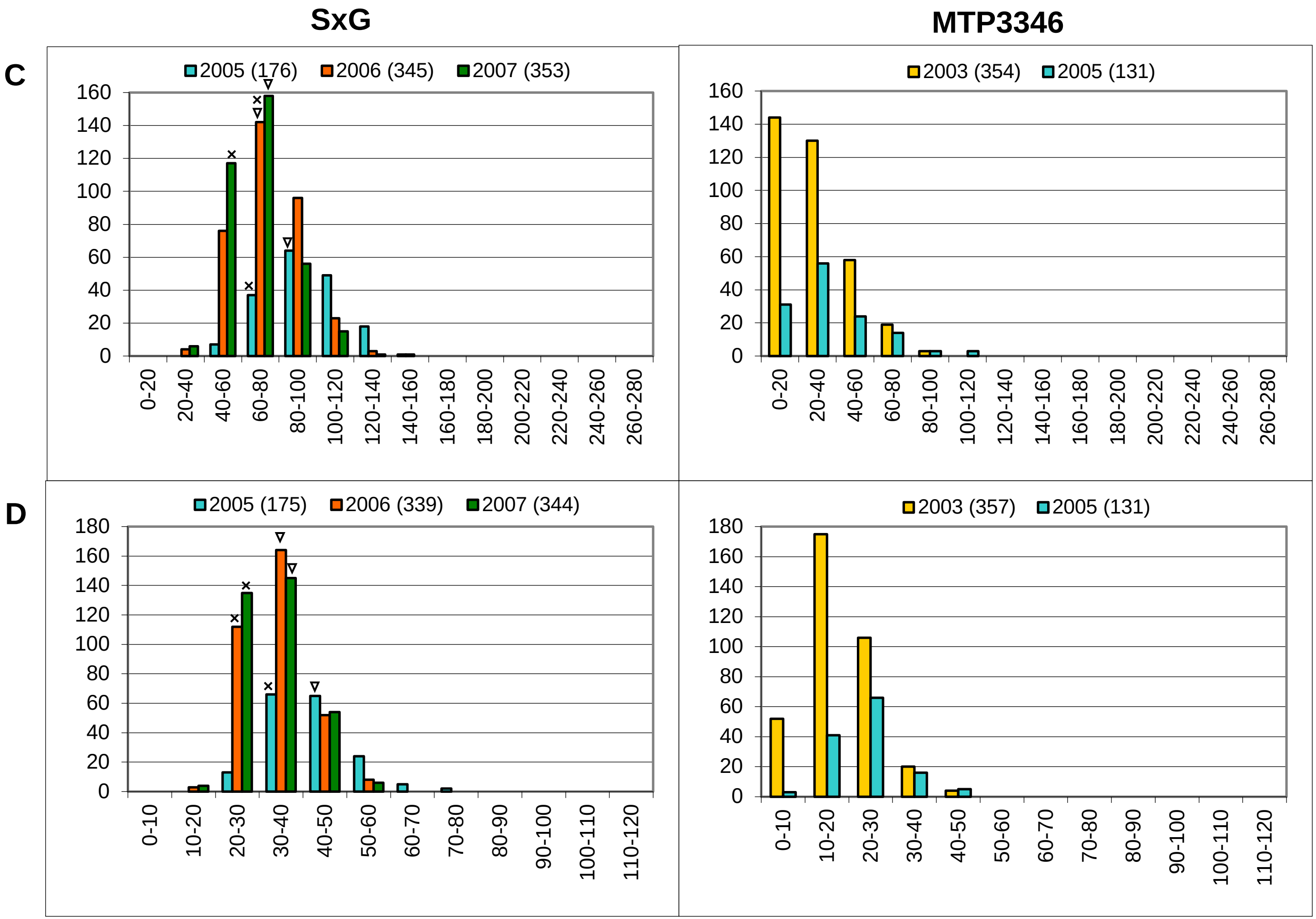

SxG
MTP3346
C
2005 (176) 2006 (345) 2007 (353)
2003 (354) 2005 (131)
D
2005 (175) 2006 (339) 2007 (344)
2003 (357) 2005 (131)
0-20
20-40
40-60
60-80
80-100
100-120
120-140
140-160
160-180
180-200
200-220
220-240
240-260
260-280
0-10
10-20
20-30
30-40
40-50
50-60
60-70
70-80
80-90
90-100
100-110
110-120

MTP3140

E

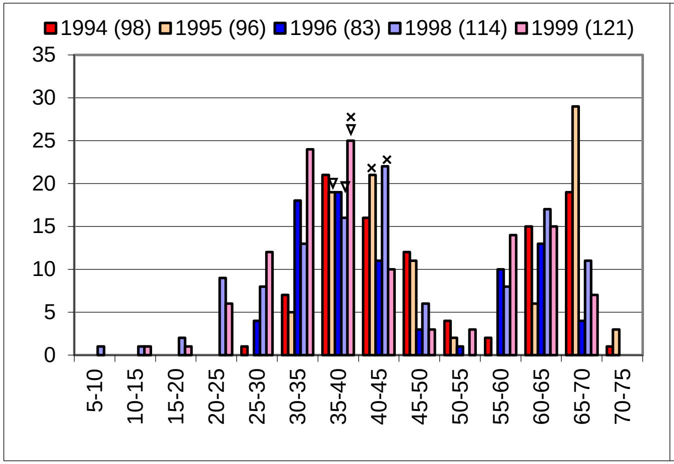

MTP3234

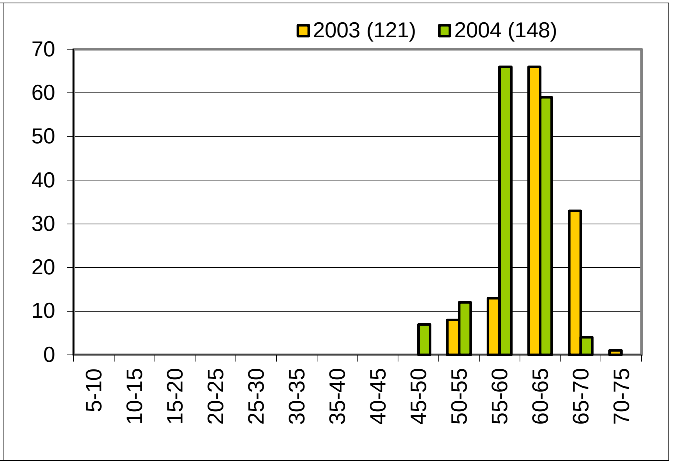

MTP3346

E

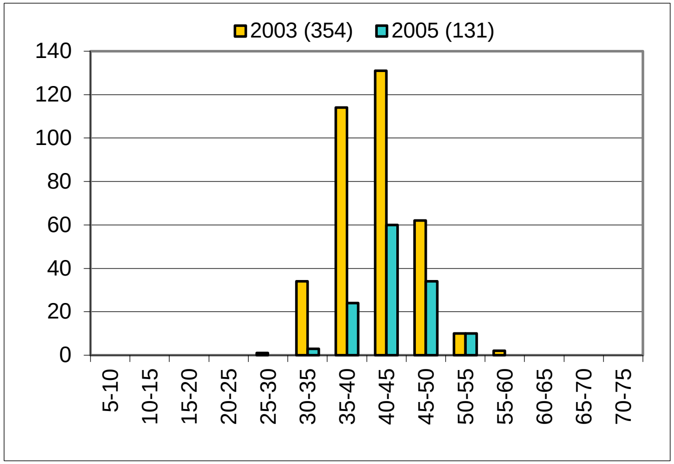

Supplement: Additional file 3: Figure S1 — Raw phenotypic data distributions for five seed and berry-related traits in four grapevine mapping populations. [file 1471-2229-13-217-S3.pdf]
