# Supplementary material for: New stable QTLs for berry weight do not colocalize with QTLs for seed traits in cultivated grapevine (Vitis vinifera L.)
Source: BMC Plant Biol. 2013 Dec 19;13:217. doi: 10.1186/1471-2229-13-217 (PMC3878267; doi:10.1186/1471-2229-13-217)
Supplement: Additional file 4: Table S3 — Transformations applied to raw data and model selected to estimate BLUPs of genetic values and broad-sense heritability, for seven seed and berry-related traits in four grapevine mapping populations. [file 1471-2229-13-217-S4.pdf]

Additional file 4: Table S3 - Transformations applied to raw data and model selected to estimate BLUPs of genetic values and broad-sense heritability, for seven seed and berry-related traits in four grapevine mapping populations.

Trait	MTP3140		MTP3234		SxG		MTP3346	
	Transfo.	Model	Transfo.	Model	Transfo.	Model[1]	Transfo.	Model
MBW	sqrt	G + Y	ln	G + Y	sqrt	G + Y	sqrt	G + Y
MSN	-	G + Y	-	G	sqrt	G + Y	none	G
TSFW	sqrt	G + Y	-	G[2]	sqrt	G + Y	sqrt	G + Y
MSFW	sqrt	G + Y	sqrt	G + Y	sqrt	G + Y	sqrt	G + Y
%SDM	ln	G + Y	none	G + Y	sqrt	-	sqrt	G + Y
RESN	sqrt(+3)	G	sqrt(+2)	G	sqrt(+2)	G	sqrt(+2)	G
RESFW	-	G	sqrt(+2)	G	sqrt(+2)	G	sqrt(+2)	G

[1] block effect was tested but never retained in model selection

[2] model without year was selected even if BIC was larger than for the model with year, because year effect was not significant

Transfo.: transformation of raw data; sqrt: square root; ln: neperian logarithm; -: not transformed

G: random genotype effect, Y: fixed year effect

MBW: mean berry weight; MSN: mean seed number; TSFW: total seed fresh weight; MSFW: mean seed fresh weight; %SDM: seed dry matter percentage; RESN: residual berry weight unexplained by seed number; RESFW: residual berry weight unexplained by total seed fresh weight
