# Supplementary material for: New stable QTLs for berry weight do not colocalize with QTLs for seed traits in cultivated grapevine (Vitis vinifera L.)
Source: BMC Plant Biol. 2013 Dec 19;13:217. doi: 10.1186/1471-2229-13-217 (PMC3878267; doi:10.1186/1471-2229-13-217)
Supplement: Additional file 5: Table S4 — Phenotypic correlations between years for seven seed and berry-related traits in four grapevine mapping populations (Spearman correlation coefficient). [file 1471-2229-13-217-S5.pdf]

Additional file 5: Table S4 - Phenotypic correlations between years for seven seed and berry-related traits in four grapevine mapping populations (Spearman correlation coefficient).

		MBW	MSN	TSFW	MSFW	%SDM	RESN	RESFW
MTP3140	1994-1995	0.84	0.84	0.97	0.91	0.89	0.75	0.67
	1994-1996	0.83	0.84	0.95	0.86	0.81	0.73	0.62
	1994-1998	0.84	0.70	0.94	0.90	0.86	0.70	0.65
	1994-1999	0.86	0.64	0.94	0.88	0.85	0.69	0.71
	1995-1996	0.81	0.82	0.95	0.92	0.82	0.77	0.61
	1995-1998	0.85	0.77	0.94	0.94	0.86	0.79	0.63
	1995-1999	0.82	0.69	0.94	0.93	0.86	0.74	0.57
	1996-1998	0.84	0.71	0.94	0.93	0.83	0.74	0.72
	1996-1999	0.83	0.60	0.94	0.90	0.79	0.67	0.63
	1998-1999	0.88	0.63	0.95	0.95	0.88	0.85	0.73
MTP3234	2002-2003	0.64	-	-	-	-	-	-
	2002-2004	0.73	-	-	-	-	-	-
	2003-2004	0.80	0.52	0.60	0.70	0.41	0.74	0.64
SxG	2005-2006	0.74	0.62	0.46	0.57	-	0.76	0.75
	2005-2007	0.76	0.76	0.58	0.68	-	0.75	0.71
	2006-2007	0.69	0.62	0.48	0.59	-	0.70	0.70
MTP3346	2003-2005	0.40	0.47	0.56	0.53	0.50	0.41	0.38

All correlations were always highly significant ($P < 0.001$).

MBW: mean berry weight; MSN: mean seed number; TSFW: total seed fresh weight; MSFW: mean seed fresh weight; %SDM: seed dry matter percentage; RESN: residual berry weight unexplained by seed number; RESFW: residual berry weight unexplained by total seed fresh weight
