# Supplementary material for: New stable QTLs for berry weight do not colocalize with QTLs for seed traits in cultivated grapevine (Vitis vinifera L.)
Source: BMC Plant Biol. 2013 Dec 19;13:217. doi: 10.1186/1471-2229-13-217 (PMC3878267; doi:10.1186/1471-2229-13-217)
Supplement: Additional file 6: Figure S2 — Genetic correlations (among BLUPs of the genetic value) between seven seed and berry-related traits within years in the grapevine mapping population MTP3346. [file 1471-2229-13-217-S6.pdf]

Additional file 6: Figure S2 - Genetic correlations (among BLUPs of the genetic value) between seven seed and berry-related traits within years in the grapevine mapping population MTP3346. Background cell color indicates Spearman test significance: alpha=5% (light yellow), 1% (dark yellow), 0.1% (orange), not significant (white). MBW: mean berry weight; MSN: mean seed number; TSFW: total seed fresh weight; MSFW: mean seed fresh weight; %SDM: seed dry matter percentage; RESN: residual berry weight unexplained by seed number; RESFW: residual berry weight unexplained by total seed fresh weight.

	MSN	TSFW	MSFW	%SDM	RESN	RESFW
MBW	0.38	0.29	0.09	0.02	0.90	0.94
MSN		0.80	0.32	0.21	0.00	0.15
TSFW			0.80	0.37	-0.02	-0.01
MSFW				0.36	-0.02	-0.16
%SDM					-0.09	-0.10
RESN						0.96

*** $P < 0.001$
** $P < 0.01$
* $P < 0.05$
