# Supplementary material for: New stable QTLs for berry weight do not colocalize with QTLs for seed traits in cultivated grapevine (Vitis vinifera L.)
Source: BMC Plant Biol. 2013 Dec 19;13:217. doi: 10.1186/1471-2229-13-217 (PMC3878267; doi:10.1186/1471-2229-13-217)
Supplement: Additional file 7: Table S5 — Broad sense heritability of seven seed and berry-related traits in the grapevine mapping population MTP3346. [file 1471-2229-13-217-S7.pdf]

Additional file 7: Table S5 - Broad sense heritability of seven seed and berry-related traits in the grapevine mapping population MTP3346.

	MBW	MSN	TSFW	MSFW	%SDM	RESN	RESFW
MTP3346	0.51	0.59	0.64	0.64	0.62	0.52	0.52

MBW: mean berry weight; MSN: mean seed number; TSFW: total seed fresh weight; MSFW: mean seed fresh weight; %SDM: seed dry matter percentage; RESN: residual berry weight unexplained by seed number; RESFW: residual berry weight unexplained by total seed fresh weight
