# Supplementary material for: New stable QTLs for berry weight do not colocalize with QTLs for seed traits in cultivated grapevine (Vitis vinifera L.)
Source: BMC Plant Biol. 2013 Dec 19;13:217. doi: 10.1186/1471-2229-13-217 (PMC3878267; doi:10.1186/1471-2229-13-217)
Supplement: Additional file 8: Table S6 — Summary of main QTLs for seven seed and berry-related traits in the grapevine mapping population MTP3346 (consensus map), derived with the MQM method. [file 1471-2229-13-217-S8.pdf]

Additional file 8: Table S6 - Summary of main QTLs for seven seed and berry-related traits in the grapevine mapping population MTP3346 (consensus map), derived with the MQM method.

**Trait**[1]	**LG**	**Population**	**Years**	**CI extremes**	**Max LOD peak**	**Max % variance**	**Largest allelic effects**[2]
%SDM	5	MTP3346	03,05	0-11.3	17.4	31	Af,Am
MSN	14	MTP3346	03,05	19.9-36.4	12.4	23	Af,Am,D

Only the QTLs found for at least two years are presented. All QTLs in this table were also significant for the BLUP of the studied trait. Confidence Interval (CI) extremes were the extremes of all CIs of both year-specific and BLUP QTLs. The genome-wide first type error rate was $\alpha = 0.05$.

[1]MSN: mean seed number; %SDM: seed dry matter percentage

[2]Major allelic effects: Af female, Am male, D dominance
