# Supplementary material for: New stable QTLs for berry weight do not colocalize with QTLs for seed traits in cultivated grapevine (Vitis vinifera L.)
Source: BMC Plant Biol. 2013 Dec 19;13:217. doi: 10.1186/1471-2229-13-217 (PMC3878267; doi:10.1186/1471-2229-13-217)
Supplement: Additional file 9: Table S7 — Summary of main QTLs for seven seed and berry-related traits in the grapevine mapping population MTP3346 (parental maps), derived with the CIM method. [file 1471-2229-13-217-S9.pdf]

Additional file 9: Table S7 - Summary of main QTLs for seven seed and berry-related traits in the grapevine mapping population MTP3346 (parental maps), derived with the CIM method.

**Trait**	**LG**	**population**	**map**	**year**	**CI extremes**	**LOD**	**%var**
%SDM	5	MTP3346	F	03,05	0.0-7.2	6.0	14

Only the QTL found for at least two years is presented. It was also significant for the BLUP. Confidence Interval (CI) extremes were the extremes of all CIs of both year-specific and BLUP QTLs. The genome-wide first type error rate was $\alpha = 0.05$.

F: female

%SDM: seed dry matter percentage
