# Supplementary material for: New stable QTLs for berry weight do not colocalize with QTLs for seed traits in cultivated grapevine (Vitis vinifera L.)
Source: BMC Plant Biol. 2013 Dec 19;13:217. doi: 10.1186/1471-2229-13-217 (PMC3878267; doi:10.1186/1471-2229-13-217)
Supplement: Additional file 10: Table S8 — Detailed features of all QTLs for seven seed and berry-related traits in four grapevine mapping populations (consensus maps), derived with the MQM method. [file 1471-2229-13-217-S10.pdf]

Additional file 10: Table S8 - Detailed features of all QTLs for seven seed and berry-related traits in four grapevine mapping populations (consensus maps), derived with the MQM method.

				QTL position (cM)		QTL effect			
**Trait**	**LG**	**Population**	**Year[1]**	**LOD peak**	**CI**	**LOD**	**LOD threshold**	**%var**	**Largest allelic effects**
MBW	1	MTP3140	B	37.4	32.7-43.4	5.3	4.8	5	Af,D
MBW	4	SxG	2005	53.7	47.7-56	5.1	4.6	7	Af,D
MBW	5	MTP3234	2002	24.0	13-32	5.5	4.7	22	Am,D
**MBW**	**8**	**SxG**	**B**	**32.7**	**24.7-56.3**	**7.0**	**4.6**	**10**	**Af**
**MBW**	**8**	**SxG**	**B**	**46.4**	**22.7-56.3**	**6.7**	**4.6**	**9**	**Af**
**MBW**	**8**	**SxG**	**2005**	**30.7**	**21.2-48.4**	**5.6**	**4.6**	**9**	**Af,D**
**MBW**	**8**	**SxG**	**2005**	**42.4**	**21.2-56.3**	**4.9**	**4.6**	**7**	**Af**
**MBW**	**8**	**SxG**	**2006**	**32.7**	**24.7-44.4**	**8.5**	**4.5**	**16**	**Af**
**MBW**	**11**	**MTP3140**	**B**	**14.9**	**9.6-24.9**	**6.6**	**4.8**	**6**	**Af,Am**
**MBW**	**11**	**MTP3140**	**1995**	**3.6**	**0-10.1**	**6.1**	**4.6**	**11**	**Am**
**MBW**	**11**	**MTP3140**	**1998**	**16.9**	**9.6-25.7**	**6.1**	**4.9**	**10**	**Af,Am**
MBW	13	SxG	B	23.6	19.1-30.8	6.0	4.6	8	Af
MBW	13	SxG	2005	26.8	19.6-32.2	6.5	4.6	10	Af,D
MBW	14	MTP3140	1999	30.0	21.9-33.7	4.9	4.7	7	Am
**MBW**	**17**	**MTP3140**	**B**	**22.9**	**21.2-28.3**	**10.3**	**4.8**	**10**	**Am,D**
**MBW**	**17**	**MTP3140**	**1995**	**22.9**	**21.2-28.3**	**8.6**	**4.6**	**13**	**Am,D**
**MBW**	**17**	**MTP3140**	**1998**	**21.2**	**18-26.3**	**7.2**	**4.9**	**11**	**Am,D**
**MBW**	**17**	**MTP3140**	**1999**	**22.9**	**21.2-28.3**	**4.9**	**4.7**	**7**	**Am**
**MBW**	**17**	**SxG**	**B**	**16.1**	**11.3-20.1**	**17.1**	**4.6**	**23**	**Am**
**MBW**	**17**	**SxG**	**2005**	**13.3**	**9.3-18.1**	**15.5**	**4.6**	**24**	**Am**
**MBW**	**17**	**SxG**	**2006**	**14.1**	**9.3-20.4**	**9.7**	**4.5**	**15**	**Am**
**MBW**	**17**	**SxG**	**2007**	**13.3**	**9.3-20.4**	**15.4**	**4.6**	**29**	**Am**
**MBW**	**17**	**SxG**	**2007**	**16.1**	**11.3-20.4**	**16.2**	**4.6**	**31**	**Am**
**MBW**	**18**	**MTP3140**	**B**	**93.0**	**87.6-93.3**	**38.6**	**4.8**	**61**	**Af,Am**
**MBW**	**18**	**MTP3140**	**1994**	**91.0**	**87.6-93.3**	**26.1**	**4.9**	**61**	**Af,Am**
**MBW**	**18**	**MTP3140**	**1995**	**93.0**	**91-93.3**	**27.4**	**4.6**	**58**	**Af,Am**
**MBW**	**18**	**MTP3140**	**1996**	**91.0**	**87.6-93.3**	**17.1**	**4.7**	**49**	**Af,Am**
**MBW**	**18**	**MTP3140**	**1998**	**93.0**	**87.6-93.3**	**22.2**	**4.9**	**45**	**Af,Am**
**MBW**	**18**	**MTP3140**	**1999**	**89.6**	**87.6-93**	**28.3**	**4.7**	**58**	**Af,Am**
**MBW**	**18**	**SxG**	**B**	**36.9**	**34-42.9**	**6.6**	**4.6**	**8**	**Am**
**MBW**	**18**	**SxG**	**2006**	**34.9**	**32.6-42.9**	**5.9**	**4.5**	**9**	**Am**
**MBW**	**18**	**SxG**	**2007**	**36.9**	**34-43.3**	**5.0**	**4.6**	**9**	**Am,D**
RESFW	5	MTP3346	B	0.0	0-7.3	4.0	3.8	5	Af
**RESFW**	**8**	**SxG**	**B**	**32.7**	**21.2-56.3**	**8.1**	**4.6**	**10**	**Af**
**RESFW**	**8**	**SxG**	**B**	**46.4**	**21.2-56.3**	**8.0**	**4.6**	**10**	**Af**
**RESFW**	**8**	**SxG**	**2005**	**30.7**	**21.2-48.4**	**5.9**	**4.6**	**9**	**Af**
**RESFW**	**8**	**SxG**	**2005**	**40.4**	**21.2-54.4**	**5.3**	**4.6**	**8**	**Af**
**RESFW**	**8**	**SxG**	**2006**	**34.7**	**26.7-52.4**	**10.6**	**4.6**	**16**	**Af**
**RESFW**	**8**	**SxG**	**2006**	**44.4**	**28.7-52.4**	**10.7**	**4.6**	**17**	**Af,Am**
**RESFW**	**8**	**SxG**	**2007**	**17.2**	**9.2-34.7**	**4.9**	**4.5**	**7**	**Af**
RESFW	11	MTP3140	1995	3.6	0-9.6	6.3	4.7	26	Am
**RESFW**	**13**	**SxG**	**B**	**6.0**	**0-19.6**	**8.5**	**4.6**	**10**	**Af,Am**
**RESFW**	**13**	**SxG**	**B**	**11.1**	**0-19.6**	**8.9**	**4.6**	**11**	**Af,Am**
**RESFW**	**13**	**SxG**	**2005**	**26.8**	**19.6-32.2**	**9.7**	**4.6**	**18**	**Af**

**RESFW**	**13**	**SxG**	**2006**	**7.1**	**0-15.1**	**6.1**	**4.6**	**8**	**Af,Am**
**RESFW**	**13**	**SxG**	**2007**	**15.1**	**6-23.6**	**7.2**	**4.5**	**10**	**Af,Am**
RESFW	17	MTP3140	B	21.2	18-26.3	4.8	4.7	15	Am
RESFW	17	MTP3140	1995	10.7	7.8-15.4	4.7	4.7	15	Am,D
**RESFW**	**17**	**SxG**	**B**	**13.3**	**9.3-20.4**	**16.3**	**4.6**	**21**	**Am**
**RESFW**	**17**	**SxG**	**B**	**16.1**	**11.3-20.1**	**16.7**	**4.6**	**22**	**Am**
**RESFW**	**17**	**SxG**	**2005**	**14.1**	**9.3-20.1**	**12.9**	**4.6**	**21**	**Am**
**RESFW**	**17**	**SxG**	**2006**	**14.1**	**9.3-20.1**	**10.8**	**4.6**	**14**	**Am**
**RESFW**	**17**	**SxG**	**2007**	**16.1**	**11.3-20.1**	**16.4**	**4.5**	**24**	**Af,Am**
**RESFW**	**18**	**SxG**	**B**	**38.9**	**34-43.3**	**9.5**	**4.6**	**12**	**Am**
**RESFW**	**18**	**SxG**	**2006**	**36.9**	**34-42.9**	**8.1**	**4.6**	**11**	**Am**
**RESFW**	**18**	**SxG**	**2007**	**40.9**	**34-43.3**	**5.7**	**4.5**	**7**	**Am**
RESFW	19	MTP3234	2003	33.0	18-41	5.7	4.6	22	Af,Am
RESN	1	SxG	B	21.1	15.7-23.9	5.7	4.6	6	Af,Am
RESN	1	SxG	2005	33.1	25.9-37.1	4.6	4.5	6	Am
RESN	7	SxG	B	55.2	52.6-57.2	4.7	4.6	5	Af,Am,D
**RESN**	**8**	**SxG**	**B**	**30.7**	**22.7-56.3**	**8.5**	**4.6**	**10**	**Af**
**RESN**	**8**	**SxG**	**B**	**48.4**	**40.4-56.3**	**9.5**	**4.6**	**11**	**Af**
**RESN**	**8**	**SxG**	**2005**	**32.7**	**21.2-56.3**	**5.5**	**4.5**	**8**	**Af**
**RESN**	**8**	**SxG**	**2005**	**48.4**	**22.7-56.3**	**6.0**	**4.5**	**9**	**Af**
**RESN**	**8**	**SxG**	**2006**	**32.7**	**24.7-46.4**	**8.3**	**4.6**	**15**	**Af**
**RESN**	**8**	**SxG**	**2006**	**40.4**	**22.7-52.4**	**7.5**	**4.6**	**13**	**Af**
**RESN**	**11**	**MTP3140**	**B**	**14.9**	**9.6-24.9**	**7.5**	**4.7**	**12**	**Af,Am**
**RESN**	**11**	**MTP3140**	**1994**	**7.6**	**0-10.1**	**7.3**	**4.7**	**15**	**Af,Am**
**RESN**	**11**	**MTP3140**	**1995**	**0.0**	**0-3.6**	**7.2**	**4.8**	**18**	**Af,Am**
**RESN**	**11**	**MTP3140**	**1998**	**20.9**	**12.1-33.7**	**6.5**	**4.7**	**13**	**Af,Am**
RESN	11	MTP3234	2003	27.0	16-37	6.5	4.6	24	Af,D
RESN	13	SxG	B	13.1	0-19.6	6.3	4.6	7	Af,Am
RESN	13	SxG	2005	26.8	19.6-32.2	7.9	4.5	13	Af,D
**RESN**	**17**	**MTP3140**	**B**	**21.2**	**18-24.9**	**8.8**	**4.7**	**13**	**Am**
**RESN**	**17**	**MTP3140**	**1994**	**19.2**	**18-26.3**	**5.1**	**4.7**	**9**	**Am**
**RESN**	**17**	**MTP3140**	**1995**	**22.9**	**21.2-30.3**	**6.4**	**4.8**	**12**	**Am**
**RESN**	**17**	**MTP3140**	**1998**	**21.2**	**18-24.9**	**7.4**	**4.7**	**13**	**Am**
**RESN**	**17**	**SxG**	**B**	**16.1**	**11.3-20.1**	**17.8**	**4.6**	**21**	**Am**
**RESN**	**17**	**SxG**	**2005**	**14.1**	**11.3-18.1**	**13.6**	**4.5**	**21**	**Am**
**RESN**	**17**	**SxG**	**2006**	**14.1**	**9.3-20.4**	**8.9**	**4.6**	**14**	**Am**
**RESN**	**17**	**SxG**	**2007**	**18.1**	**13.3-22.4**	**15.1**	**4.7**	**29**	**Am**
**RESN**	**18**	**MTP3140**	**B**	**89.6**	**85.6-93.3**	**21.1**	**4.7**	**39**	**Af,Am**
**RESN**	**18**	**MTP3140**	**1994**	**91.0**	**87.6-93.3**	**15.1**	**4.7**	**32**	**Am,D**
**RESN**	**18**	**MTP3140**	**1995**	**89.6**	**85.6-93.3**	**19.3**	**4.8**	**41**	**Af,Am,D**
**RESN**	**18**	**MTP3140**	**1996**	**89.6**	**84-93.3**	**6.8**	**4.7**	**24**	**Am,D**
**RESN**	**18**	**MTP3140**	**1998**	**89.6**	**85.6-93.3**	**14.5**	**4.7**	**30**	**Af,Am**
**RESN**	**18**	**MTP3140**	**1999**	**91.0**	**87.6-93**	**16.5**	**4.8**	**45**	**Af,Am**
**RESN**	**18**	**SxG**	**B**	**36.9**	**34-43.3**	**7.2**	**4.6**	**7**	**Am**
**RESN**	**18**	**SxG**	**2006**	**34.9**	**32.6-40.9**	**6.3**	**4.6**	**10**	**Am**
**RESN**	**18**	**SxG**	**2007**	**34.9**	**34-42.9**	**5.8**	**4.7**	**10**	**Am**
RESN	19	MTP3234	2003	30.0	14-37	8.2	4.6	25	Af

MSN	2	SxG	B	12.0	6-18	17.5	4.6	37	Am,D
MSN	2	SxG	2005	14.0	8-21.3	11.1	4.6	30	Am,D
MSN	2	SxG	2006	12.0	2-23.3	5.5	4.7	12	Am,D
MSN	2	SxG	2007	12.0	6-18	24.8	4.7	48	Af,Am,D
MSN	4	SxG	B	53.7	49.7-56	13.9	4.6	24	Af
MSN	4	SxG	2005	56.0	49.7-56	8.1	4.6	15	Af
MSN	4	SxG	2006	56.0	51.7-56	15.8	4.7	29	Af
MSN	4	SxG	2007	53.7	47.7-56	9.0	4.7	14	Af
MSN	8	MTP3346	B	16.5	6-16.5	3.7	3.7	4	Am,D
MSN	14	MTP3234	B	28.0	19-34	7.4	4.5	25	Af,Am
MSN	14	MTP3234	B	22.0	19-36	6.6	4.5	19	Af,Am
MSN	14	MTP3234	2004	30.0	24-36	8.2	4.6	28	Af,Am
MSN	14	MTP3234	2004	22.0	19-37	6.4	4.6	19	Af,Am
MSN	14	MTP3346	B	29.9	23.9-35.9	12.4	3.7	19	Am,D
MSN	14	MTP3346	2003	29.9	23.9-35.9	9.9	4.0	17	Am,D
MSN	14	MTP3346	2005	27.9	19.9-36.4	5.2	3.9	23	Af,D
MSN	18	MTP3140	B	93.3	89.6-94.9	24.4	6.3	57	Af,D
MSN	18	MTP3140	1994	98.3	96.5-98.3	21.3	9.5	59	Af,Am
MSN	18	MTP3140	1998	94.9	93-96.5	17.4	4.8	51	Af,Am,D
MSN	18	MTP3140	1999	93.3	89.6-94.9	8.2	4.7	27	Af,D
TSFW	1	SxG	2006	37.1	31.9-39.3	4.7	4.6	9	Af,Am
TSFW	4	SxG	B	56.0	51.7-56	12.8	4.7	27	Af
TSFW	4	SxG	2006	56.0	51.7-56	14.2	4.6	29	Af
TSFW	4	SxG	2007	56.0	51.7-56	9.8	4.6	22	Af
TSFW	5	MTP3346	B	7.3	2-13.3	10.1	3.7	11	Af,Am
TSFW	5	MTP3346	2003	6.0	2-11.3	10.9	3.8	13	Af,Am
TSFW	8	MTP3140	1998	64.3	56.3-69	5.6	5.0	3	Af,Am
TSFW	12	MTP3346	B	24.6	14.6-34.3	7.1	3.7	10	Af,Am
TSFW	12	MTP3346	2003	24.6	14-34.3	5.7	3.8	8	Af,Am
TSFW	14	MTP3234	B	28.0	24-34	11.0	4.7	36	Af,Am,D
TSFW	14	MTP3234	2004	30.0	24-34	12.8	4.5	41	Af,D
TSFW	14	MTP3346	B	27.9	21.9-33.9	12.6	3.7	19	D
TSFW	14	MTP3346	2003	27.9	23.9-33.9	12.2	3.8	19	D
TSFW	18	MTP3140	B	93.0	91-93.3	45.6	6.8	80	Af,Am
TSFW	18	MTP3140	1996	93.0	91-93.3	32.5	7.5	75	Af,Am
TSFW	18	MTP3140	1998	93.0	91-93.3	45.4	5.0	82	Af,Am
TSFW	18	MTP3140	1999	91.0	89.6-93	40.6	5.7	78	Af,Am
MSFW	1	SxG	B	21.1	17.7-31.9	8.0	4.6	13	Af,Am
MSFW	1	SxG	B	25.9	17.7-31.9	7.7	4.6	13	Af,Am
MSFW	1	SxG	2006	23.1	19.1-33.1	6.7	4.7	15	Af,Am
MSFW	1	SxG	2006	27.9	19.1-33.1	6.6	4.7	15	Af,Am
MSFW	1	SxG	2007	17.7	14-23.9	5.8	4.4	9	Af,Am
MSFW	1	SxG	2007	21.1	14-23.9	5.8	4.4	9	Af,Am
MSFW	2	SxG	B	10.0	4-18	18.3	4.6	40	Am,D
MSFW	2	SxG	2005	12.0	4-18	11.3	4.6	35	Am
MSFW	2	SxG	2006	10.0	2-20	7.8	4.7	21	Am,D
MSFW	2	SxG	2006	25.3	0-33.3	5.7	4.7	13	Am,D

**MSFW**	**2**	**SxG**	**2007**	**10.0**	**4-16**	**21.4**	**4.4**	**45**	**Am,D**
MSFW	5	MTP3346	B	6.0	0-11.3	10.8	3.8	13	Af,Am
MSFW	5	MTP3346	2003	6.0	0-11.3	10.2	3.9	13	Af,Am
MSFW	11	MTP3234	2004	35.0	25-62	4.7	4.6	14	Af,Am
**MSFW**	**12**	**MTP3140**	**B**	**14.1**	**4.1-21**	**6.5**	**4.7**	**2**	**Am,D**
**MSFW**	**12**	**MTP3140**	**1995**	**4.1**	**0-15**	**6.7**	**4.6**	**3**	**Am,D**
**MSFW**	**12**	**MTP3140**	**1999**	**15.0**	**2.1-23**	**5.7**	**5.1**	**3**	**Am**
MSFW	12	MTP3346	B	30.6	20.6-34.3	6.3	3.8	8	Af,Am
MSFW	12	MTP3346	2003	30.6	18.6-34.3	6.0	3.9	8	Af,Am
MSFW	14	MTP3140	1995	47.5	46.1-52.5	5.7	4.6	2	Am
MSFW	14	MTP3234	B	32.0	26-36	7.8	4.5	30	Af,D
MSFW	14	MTP3234	2004	30.0	26-36	8.3	4.6	27	Af,D
MSFW	14	MTP3346	B	12.0	0-33.9	4.0	3.8	6	D
MSFW	14	MTP3346	2003	27.9	19.9-35.9	4.4	3.9	7	D
MSFW	16	MTP3234	2003	12.0	8-17	5.5	4.6	19	Af,Am
**MSFW**	**18**	**MTP3140**	**B**	**93.0**	**91-93.3**	**61.4**	**4.7**	**87**	**Af,Am**
**MSFW**	**18**	**MTP3140**	**1994**	**91.0**	**87.6-93**	**31.5**	**5.3**	**76**	**Af,Am**
**MSFW**	**18**	**MTP3140**	**1995**	**91.0**	**87.6-93**	**9.7**	**4.6**	**1**	**Af,D**
**MSFW**	**18**	**MTP3140**	**1995**	**98.3**	**96.5-98.3**	**9.5**	**4.6**	**8**	**Am,D**
**MSFW**	**18**	**MTP3140**	**1998**	**93.0**	**91-93.3**	**46.3**	**4.9**	**87**	**Af,Am**
**MSFW**	**18**	**MTP3140**	**1999**	**91.0**	**89.6-93**	**55.8**	**5.1**	**84**	**Af,Am**
%SDM	4	MTP3346	B	14.0	4-15.9	4.1	3.8	5	Af,Am
%SDM	4	MTP3346	2003	14.0	4-15.9	4.7	3.8	6	Af,Am
**%SDM**	**5**	**MTP3346**	**B**	**2.0**	**0-6**	**17.4**	**3.8**	**20**	**Af,Am**
**%SDM**	**5**	**MTP3346**	**2003**	**2.0**	**0-6**	**13.8**	**3.8**	**17**	**Af,Am**
**%SDM**	**5**	**MTP3346**	**2005**	**2.0**	**0-6**	**9.3**	**4.0**	**31**	**Af,Am**
**%SDM**	**14**	**MTP3234**	**B**	**37.0**	**24-41**	**12.3**	**4.6**	**46**	**Am,D**
**%SDM**	**14**	**MTP3234**	**B**	**32.0**	**28-36**	**16.1**	**4.6**	**51**	**Am,D**
**%SDM**	**14**	**MTP3234**	**2003**	**32.0**	**26-36**	**6.3**	**4.9**	**35**	**Am,D**
**%SDM**	**14**	**MTP3234**	**2004**	**32.0**	**26-36**	**13.0**	**4.7**	**46**	**Am,D**
**%SDM**	**18**	**MTP3140**	**B**	**93.0**	**91-93.3**	**47.9**	**4.6**	**84**	**Af,Am**
**%SDM**	**18**	**MTP3140**	**1998**	**89.6**	**87.6-91**	**11.5**	**4.7**	**5**	**Af,Am**
**%SDM**	**18**	**MTP3140**	**1998**	**93.0**	**87.6-93.3**	**10.4**	**4.7**	**3**	**Af,Am**
**%SDM**	**18**	**MTP3140**	**1999**	**91.0**	**87.6-93**	**33.3**	**5.2**	**35**	**Af,Am**

QTLs present in at least two years are outlined in bold.

MBW: mean berry weight; MSN: mean seed number; TSFW: total seed fresh weight; MSFW: mean seed fresh weight; %SDM: seed dry matter percentage; RESN: residual berry weight unexplained by seed number; RESFW: residual berry weight unexplained by total seed fresh weight

CI: confidence interval

Major allelic effects: Af female, Am male, D dominance

[1] B stands for inter-year BLUP
