# Supplementary material for: New stable QTLs for berry weight do not colocalize with QTLs for seed traits in cultivated grapevine (Vitis vinifera L.)
Source: BMC Plant Biol. 2013 Dec 19;13:217. doi: 10.1186/1471-2229-13-217 (PMC3878267; doi:10.1186/1471-2229-13-217)
Supplement: Additional file 11: Table S9 — Detailed features of all QTLs for seven seed and berry-related traits in four grapevine mapping populations (parental maps), derived with the CIM method. [file 1471-2229-13-217-S11.pdf]

Additional file 11: Table S9 - Detailed features of all QTLs for seven seed and berry-related traits in four grapevine mapping populations (parental maps), derived with the CIM method.

					QTL position (cM)		QTL effect		
**Trait**	**LG**	**Population**	**Map**	**Year**	**LOD peak**	**CI**	**LOD**	**LOD threshold**	**%var**
MBW	1	SG	M	2006	16.6	12.11 - 22.61	4.3	3.7	6.4
**MBW**	**8**	**SG**	**F**	**2005**	**17.1**	**6.01 - 27.11**	**7.7**	**3.8**	**16.5**
**MBW**	**8**	**SG**	**F**	**2006**	**23.1**	**17.11 - 33.41**	**9.4**	**3.6**	**20.3**
**MBW**	**8**	**SG**	**F**	**2006**	**29.4**	**15.11 - 37.41**	**8.6**	**3.6**	**17.1**
**MBW**	**8**	**SG**	**F**	**2007**	**8.0**	**2.01 - 27.11**	**5.3**	**3.6**	**12.5**
**MBW**	**8**	**SG**	**F**	**2007**	**19.1**	**0.01 - 27.11**	**5.0**	**3.6**	**11.4**
MBW	8	MTP3346	F	2005	14.6	11.61 - 14.61	2.7	2.6	11.7
MBW	12	SG	F	2005	6.0	0.01 - 14.01	4.9	3.8	14.7
**MBW**	**13**	**SG**	**F**	**B**	**0.0**	**0.01 - 12.01**	**5.9**	**3.6**	**16.5**
**MBW**	**13**	**SG**	**F**	**2005**	**4.0**	**0.01 - 13.31**	**4.4**	**3.8**	**10.8**
**MBW**	**13**	**SG**	**F**	**2006**	**0.0**	**0.01 - 12.01**	**4.9**	**3.6**	**12.8**
**MBW**	**17**	**SG**	**M**	**B**	**15.7**	**10.91 - 18.31**	**15.0**	**3.8**	**25.4**
**MBW**	**17**	**SG**	**M**	**2005**	**14.7**	**9.91 - 18.31**	**10.8**	**3.7**	**19.1**
**MBW**	**17**	**SG**	**M**	**2006**	**13.7**	**9.91 - 18.31**	**8.9**	**3.7**	**14.1**
**MBW**	**17**	**SG**	**M**	**2007**	**16.7**	**10.91 - 18.31**	**14.2**	**3.8**	**24.0**
MBW	18	MTP3140	F	B	55.0	50.99 - 56.59	16.3	3.9	37.4
MBW	18	MTP3140	M	B	108.0	101.99 - 110.69	12.3	4.0	28.4
MBW	18	SG	M	B	38.2	24.21 - 49.71	4.5	3.8	7.1
MBW	18	SG	M	B	43.4	24.21 - 49.71	4.5	3.8	7.1
MBW	18	SG	M	2006	38.2	25.21 - 48.41	4.9	3.7	8.3
MBW	18	SG	M	2006	42.4	26.21 - 48.41	4.9	3.7	7.9
**MBW**	**18**	**MTP3140**	**F**	**1994**	**55.0**	**50.99 - 56.59**	**13.7**	**4.2**	**34.2**
**MBW**	**18**	**MTP3140**	**F**	**1995**	**55.0**	**50.99 - 56.59**	**11.1**	**4.2**	**27.9**
**MBW**	**18**	**MTP3140**	**F**	**1996**	**55.0**	**50.99 - 58.19**	**8.8**	**4.1**	**25.5**
**MBW**	**18**	**MTP3140**	**F**	**1998**	**55.0**	**50.99 - 56.59**	**11.7**	**4.1**	**31.0**
**MBW**	**18**	**MTP3140**	**F**	**1999**	**55.0**	**50.99 - 56.59**	**11.9**	**4.2**	**29.6**
**MBW**	**18**	**MTP3140**	**M**	**1994**	**108.0**	**103.99 - 110.69**	**12.7**	**4.1**	**32.2**
**MBW**	**18**	**MTP3140**	**M**	**1995**	**115.3**	**113.49 - 115.29**	**9.7**	**4.0**	**23.6**
**MBW**	**18**	**MTP3140**	**M**	**1996**	**106.0**	**101.49 - 110.69**	**7.7**	**4.3**	**24.6**
**MBW**	**18**	**MTP3140**	**M**	**1998**	**115.3**	**113.49 - 115.29**	**10.4**	**4.4**	**28.5**
**MBW**	**18**	**MTP3140**	**M**	**1999**	**106.0**	**101.49 - 110.69**	**10.4**	**4.2**	**27.0**
RESFW	5	MTP3346	F	B	3.0	0.01 - 7.21	4.0	2.4	5.7
RESFW	5	MTP3346	F	2003	3.0	0.01 - 12.21	2.6	2.5	3.8
**RESFW**	**8**	**SG**	**F**	**B**	**8.0**	**2.01 - 27.11**	**8.4**	**3.6**	**17.2**
**RESFW**	**8**	**SG**	**F**	**B**	**19.1**	**2.01 - 27.11**	**8.4**	**3.6**	**16.7**
**RESFW**	**8**	**SG**	**F**	**2005**	**17.1**	**12.01 - 33.41**	**6.6**	**3.8**	**14.0**
**RESFW**	**8**	**SG**	**F**	**2006**	**21.1**	**15.11 - 27.11**	**8.3**	**3.7**	**19.6**
**RESFW**	**8**	**SG**	**F**	**2007**	**8.0**	**2.01 - 25.11**	**6.2**	**3.6**	**13.5**
**RESFW**	**8**	**SG**	**F**	**2007**	**17.1**	**0.01 - 27.11**	**5.7**	**3.6**	**11.6**
RESFW	8	MTP3346	F	2005	14.6	11.61 - 14.61	2.8	2.6	12.0
RESFW	11	MTP3140	M	1998	20.2	8.19 - 27.49	4.3	4.1	11.2
RESFW	11	MTP3346	M	B	2.0	0.01 - 7.01	3.0	2.5	5.4
RESFW	11	MTP3346	M	2003	0.0	0.01 - 7.01	2.8	2.4	3.8
**RESFW**	**13**	**SG**	**F**	**B**	**0.0**	**0.01 - 8.01**	**5.5**	**3.6**	**13.6**
**RESFW**	**13**	**SG**	**F**	**2005**	**0.0**	**0.01 - 12.01**	**4.2**	**3.8**	**11.8**

**RESFW**	**13**	**SG**	**F**	**2007**	**0.0**	**0.01 - 8.01**	**4.2**	**3.6**	**11.8**
**RESFW**	**17**	**SG**	**M**	**B**	**15.7**	**9.91 - 18.31**	**15.3**	**3.7**	**23.5**
**RESFW**	**17**	**SG**	**M**	**2005**	**16.7**	**10.91 - 18.31**	**10.6**	**3.9**	**20.0**
**RESFW**	**17**	**SG**	**M**	**2006**	**13.7**	**9.91 - 18.31**	**9.4**	**3.6**	**14.8**
**RESFW**	**17**	**SG**	**M**	**2007**	**15.7**	**9.91 - 18.31**	**15.0**	**3.8**	**24.2**
**RESFW**	**17**	**MTP3140**	**M**	**B**	**23.1**	**17.51 - 25.21**	**5.7**	**4.1**	**14.6**
**RESFW**	**17**	**MTP3140**	**M**	**1994**	**25.1**	**19.51 - 29.21**	**5.0**	**4.3**	**14.5**
**RESFW**	**17**	**MTP3140**	**M**	**1995**	**25.2**	**19.51 - 31.21**	**4.8**	**4.1**	**14.0**
**RESFW**	**17**	**MTP3140**	**M**	**1998**	**23.1**	**17.51 - 25.21**	**4.4**	**4.1**	**12.1**
**RESFW**	**17**	**MTP3140**	**M**	**1999**	**23.1**	**16.61 - 25.21**	**4.5**	**4.5**	**13.1**
**RESFW**	**18**	**SG**	**M**	**B**	**36.2**	**24.21 - 49.41**	**5.6**	**3.7**	**8.7**
**RESFW**	**18**	**SG**	**M**	**B**	**43.4**	**24.21 - 49.41**	**5.7**	**3.7**	**8.2**
**RESFW**	**18**	**SG**	**M**	**2005**	**39.2**	**26.21 - 49.71**	**4.6**	**3.9**	**8.6**
**RESFW**	**18**	**SG**	**M**	**2005**	**43.4**	**27.21 - 49.71**	**4.6**	**3.9**	**8.4**
**RESFW**	**18**	**SG**	**M**	**2006**	**36.2**	**25.21 - 49.41**	**5.6**	**3.6**	**9.9**
**RESFW**	**18**	**SG**	**M**	**2006**	**43.4**	**25.21 - 49.41**	**5.6**	**3.6**	**9.2**
**RESFW**	**18**	**SG**	**M**	**2007**	**44.4**	**29.21 - 49.71**	**5.4**	**3.8**	**8.3**
RESFW	19	MTP3234	F	B	44.0	24.01 - 50.91	3.8	3.6	9.3
RESFW	19	MTP3234	F	2003	50.9	44.01 - 56.91	6.1	3.7	18.4
**RESN**	**1**	**SG**	**M**	**B**	**16.6**	**12.11 - 22.91**	**4.4**	**3.7**	**5.9**
**RESN**	**1**	**SG**	**M**	**2005**	**25.9**	**16.61 - 41.91**	**4.3**	**3.6**	**7.1**
**RESN**	**1**	**SG**	**M**	**2005**	**31.1**	**21.61 - 39.21**	**4.9**	**3.6**	**8.6**
**RESN**	**1**	**SG**	**M**	**2006**	**16.6**	**12.11 - 22.61**	**4.1**	**3.7**	**6.1**
RESN	4	MTP3140	F	1994	33.0	28.79 - 38.79	4.2	4.1	11.1
RESN	5	MTP3346	F	B	3.0	0.01 - 7.21	3.3	2.4	4.7
RESN	7	MTP3234	F	2003	13.7	11.71 - 19.71	4.3	3.7	14.9
**RESN**	**8**	**SG**	**F**	**B**	**10.0**	**2.01 - 27.11**	**8.7**	**3.8**	**18.0**
**RESN**	**8**	**SG**	**F**	**B**	**19.1**	**4.01 - 27.11**	**8.8**	**3.8**	**18.3**
**RESN**	**8**	**SG**	**F**	**2005**	**10.0**	**2.01 - 25.11**	**7.1**	**3.6**	**15.4**
**RESN**	**8**	**SG**	**F**	**2005**	**15.1**	**2.01 - 27.11**	**6.8**	**3.6**	**14.1**
**RESN**	**8**	**SG**	**F**	**2005**	**29.4**	**0.01 - 39.41**	**5.5**	**3.6**	**11.8**
**RESN**	**8**	**SG**	**F**	**2006**	**23.1**	**15.11 - 35.41**	**8.7**	**3.7**	**19.5**
**RESN**	**8**	**SG**	**F**	**2006**	**29.4**	**15.11 - 37.41**	**8.0**	**3.7**	**16.7**
**RESN**	**8**	**SG**	**F**	**2007**	**8.0**	**0.01 - 27.41**	**4.7**	**3.5**	**11.0**
**RESN**	**8**	**SG**	**F**	**2007**	**19.1**	**0.01 - 27.41**	**4.7**	**3.5**	**10.7**
RESN	8	MTP3346	F	2005	14.6	11.61 - 14.61	2.8	2.6	12.0
RESN	11	MTP3234	F	B	12.0	2.01 - 24.11	5.5	3.6	16.5
RESN	11	MTP3234	F	2004	14.0	4.01 - 24.11	5.2	3.6	16.2
**RESN**	**11**	**MTP3140**	**F**	**B**	**14.9**	**10.09 - 22.89**	**5.1**	**3.9**	**12.9**
**RESN**	**11**	**MTP3140**	**F**	**1994**	**14.9**	**10.09 - 22.89**	**4.8**	**4.1**	**12.8**
**RESN**	**11**	**MTP3140**	**F**	**1994**	**11.4**	**10.09 - 22.89**	**5.0**	**4.1**	**13.6**
**RESN**	**11**	**MTP3140**	**F**	**1996**	**16.9**	**11.39 - 22.89**	**6.0**	**4.1**	**19.8**
RESN	11	MTP3346	M	B	2.0	0.01 - 8.01	2.8	2.5	5.0
RESN	11	MTP3346	M	2003	1.0	0.01 - 7.01	2.8	2.5	4.4
RESN	13	SG	F	B	0.0	0.01 - 10.01	4.3	3.8	11.3
RESN	13	SG	F	2006	0.0	0.01 - 12.01	4.2	3.7	11.9
**RESN**	**17**	**SG**	**M**	**B**	**14.7**	**9.91 - 18.31**	**14.3**	**3.7**	**22.6**
**RESN**	**17**	**SG**	**M**	**2005**	**12.9**	**8.01 - 18.31**	**9.4**	**3.6**	**18.9**

**RESN**	**17**	**SG**	**M**	**2005**	**15.7**	**8.01 - 18.31**	**9.5**	**3.6**	**19.4**
**RESN**	**17**	**SG**	**M**	**2006**	**13.7**	**9.91 - 18.31**	**8.6**	**3.7**	**13.7**
**RESN**	**17**	**SG**	**M**	**2007**	**15.7**	**9.91 - 18.31**	**13.9**	**3.6**	**23.2**
**RESN**	**17**	**MTP3140**	**M**	**B**	**25.1**	**19.51 - 31.21**	**6.2**	**4.1**	**14.0**
**RESN**	**17**	**MTP3140**	**M**	**1994**	**25.2**	**21.11 - 31.21**	**5.1**	**4.1**	**13.6**
**RESN**	**17**	**MTP3140**	**M**	**1995**	**25.2**	**21.11 - 31.21**	**6.0**	**4.1**	**14.8**
**RESN**	**17**	**MTP3140**	**M**	**1998**	**23.1**	**17.51 - 25.21**	**5.7**	**4.3**	**15.5**
RESN	18	SG	M	B	32.2	20.21 - 47.41	5.0	3.7	8.9
RESN	18	SG	M	2006	37.2	25.21 - 47.41	5.2	3.7	9.1
RESN	18	SG	M	2006	42.4	24.21 - 48.41	5.2	3.7	8.3
**RESN**	**18**	**MTP3140**	**F**	**B**	**55.0**	**50.99 - 56.59**	**5.3**	**3.9**	**12.4**
**RESN**	**18**	**MTP3140**	**F**	**1995**	**70.9**	**60.89 - 76.89**	**4.4**	**4.1**	**14.4**
**RESN**	**18**	**MTP3140**	**F**	**1998**	**55.0**	**50.99 - 56.59**	**4.6**	**4.1**	**11.8**
**RESN**	**18**	**MTP3140**	**F**	**1999**	**55.0**	**50.99 - 58.19**	**8.2**	**4.1**	**22.4**
**RESN**	**18**	**MTP3140**	**M**	**B**	**106.0**	**101.49 - 111.49**	**8.1**	**4.1**	**19.0**
**RESN**	**18**	**MTP3140**	**M**	**1994**	**106.0**	**101.49 - 111.49**	**5.1**	**4.1**	**13.8**
**RESN**	**18**	**MTP3140**	**M**	**1995**	**102.0**	**101.49 - 110.69**	**7.0**	**4.1**	**16.9**
**RESN**	**18**	**MTP3140**	**M**	**1998**	**108.7**	**101.99 - 111.49**	**7.2**	**4.3**	**19.5**
**RESN**	**18**	**MTP3140**	**M**	**1999**	**111.5**	**96.89 - 115.29**	**7.6**	**4.2**	**19.2**
**RESN**	**18**	**MTP3140**	**M**	**1999**	**106.0**	**97.89 - 110.69**	**8.7**	**4.2**	**22.4**
**RESN**	**18**	**MTP3140**	**M**	**1999**	**99.9**	**96.89 - 115.29**	**8.2**	**4.2**	**20.3**
**RESN**	**18**	**MTP3140**	**M**	**1999**	**94.9**	**89.89 - 115.29**	**6.6**	**4.2**	**18.2**
RESN	19	MTP3234	F	2003	44.9	30.01 - 50.91	6.1	3.7	17.6
MSN	2	SG	F	B	16.0	6.01 - 25.71	5.2	3.6	12.4
MSN	2	SG	F	2007	12.0	4.01 - 21.71	10.1	3.7	22.6
MSN	2	SG	M	B	11.0	4.01 - 18.01	6.9	3.7	19.4
**MSN**	**2**	**SG**	**M**	**2005**	**17.0**	**9.01 - 24.91**	**4.7**	**3.6**	**15.3**
**MSN**	**2**	**SG**	**M**	**2005**	**20.9**	**8.01 - 25.91**	**4.5**	**3.6**	**10.1**
**MSN**	**2**	**SG**	**M**	**2007**	**12.0**	**5.01 - 20.01**	**7.8**	**3.7**	**20.0**
**MSN**	**4**	**SG**	**F**	**B**	**48.3**	**40.31 - 48.31**	**13.6**	**3.6**	**25.7**
**MSN**	**4**	**SG**	**F**	**2005**	**48.3**	**42.31 - 48.31**	**8.5**	**3.7**	**18.9**
**MSN**	**4**	**SG**	**F**	**2006**	**48.3**	**44.31 - 48.31**	**6.5**	**3.7**	**11.4**
**MSN**	**4**	**SG**	**F**	**2007**	**46.3**	**40.31 - 48.31**	**9.1**	**3.7**	**17.8**
MSN	5	MTP3140	M	1994	39.3	35.49 - 46.09	4.7	4.1	12.1
MSN	8	MTP3234	M	2004	64.4	54.71 - 80.31	4.2	3.6	11.2
MSN	8	MTP3234	M	2004	68.3	54.71 - 80.31	4.2	3.6	11.3
MSN	13	SG	M	B	21.6	12.61 - 30.41	3.9	3.7	9.7
MSN	14	MTP3234	F	B	25.5	16.01 - 27.11	3.7	3.5	9.2
MSN	14	MTP3346	M	B	28.0	16.01 - 31.01	3.8	2.5	5.4
MSN	14	MTP3346	M	2003	26.0	13.01 - 31.01	3.2	2.5	4.8
**MSN**	**18**	**MTP3140**	**F**	**B**	**53.0**	**50.99 - 58.19**	**11.5**	**3.9**	**27.9**
**MSN**	**18**	**MTP3140**	**F**	**1994**	**55.0**	**50.99 - 58.19**	**11.4**	**4.0**	**28.8**
**MSN**	**18**	**MTP3140**	**F**	**1995**	**53.0**	**50.99 - 58.19**	**9.9**	**4.0**	**26.9**
**MSN**	**18**	**MTP3140**	**F**	**1996**	**64.9**	**40.79 - 72.89**	**7.6**	**4.1**	**26.0**
**MSN**	**18**	**MTP3140**	**F**	**1996**	**55.0**	**50.99 - 58.19**	**10.0**	**4.1**	**26.4**
**MSN**	**18**	**MTP3140**	**F**	**1996**	**46.7**	**42.69 - 60.59**	**8.3**	**4.1**	**26.5**
**MSN**	**18**	**MTP3140**	**F**	**1998**	**53.0**	**50.69 - 58.19**	**4.0**	**4.0**	**11.8**
**MSN**	**18**	**MTP3140**	**M**	**B**	**115.3**	**113.49 - 115.29**	**4.9**	**4.2**	**11.7**

**MSN**	**18**	**MTP3140**	**M**	**1994**	**115.3**	**113.49 - 115.29**	**6.5**	**4.1**	**17.5**
**MSN**	**18**	**MTP3140**	**M**	**1996**	**115.3**	**113.49 - 115.29**	**4.4**	**4.2**	**13.0**
**MSN**	**18**	**MTP3140**	**M**	**1998**	**115.3**	**113.49 - 115.29**	**4.7**	**4.1**	**12.5**
MSN	18	MTP3346	F	B	22.0	13.01 - 25.01	2.7	2.5	4.5
TSFW	1	SG	F	B	39.8	36.81 - 47.81	4.4	3.7	7.1
TSFW	1	SG	F	2006	38.6	31.21 - 39.81	4.0	3.7	6.6
TSFW	2	SG	F	2007	21.7	12.01 - 27.71	4.3	3.7	7.1
TSFW	4	SG	F	B	48.3	44.31 - 48.31	16.5	3.7	31.7
**TSFW**	**4**	**SG**	**F**	**2005**	**48.3**	**42.31 - 48.31**	**5.0**	**3.6**	**12.1**
**TSFW**	**4**	**SG**	**F**	**2006**	**48.3**	**44.31 - 48.31**	**12.0**	**3.7**	**22.6**
**TSFW**	**4**	**SG**	**F**	**2007**	**48.3**	**44.31 - 48.31**	**12.4**	**3.7**	**24.0**
TSFW	5	MTP3346	F	B	7.0	0.01 - 10.21	4.0	2.4	5.5
TSFW	5	MTP3346	F	B	17.2	14.21 - 18.11	3.0	2.4	4.8
TSFW	5	MTP3346	F	2003	7.2	1.01 - 10.21	5.7	2.4	8.0
TSFW	5	MTP3346	F	2003	17.2	15.21 - 18.11	3.3	2.4	5.3
TSFW	8	MTP3234	M	B	92.1	84.11 - 92.11	4.0	3.7	9.4
**TSFW**	**13**	**SG**	**M**	**2005**	**34.4**	**30.21 - 46.41**	**4.7**	**3.7**	**11.3**
**TSFW**	**13**	**SG**	**M**	**2007**	**27.2**	**13.61 - 30.41**	**4.1**	**3.7**	**8.7**
TSFW	14	MTP3234	F	B	29.1	22.01 - 37.11	4.8	3.6	12.1
TSFW	14	MTP3234	F	2004	25.5	22.01 - 37.11	5.5	3.6	14.7
TSFW	14	MTP3234	F	2004	31.1	22.01 - 37.11	5.6	3.6	16.4
TSFW	16	MTP3234	F	B	20.6	4.79 - 34.59	5.1	3.6	19.8
TSFW	16	MTP3234	F	2003	10.8	4.79 - 32.59	4.4	3.8	13.1
**TSFW**	**18**	**MTP3140**	**F**	**B**	**55.0**	**50.99 - 56.59**	**20.3**	**4.2**	**43.7**
**TSFW**	**18**	**MTP3140**	**F**	**1994**	**55.0**	**50.99 - 56.59**	**18.4**	**4.2**	**42.2**
**TSFW**	**18**	**MTP3140**	**F**	**1995**	**55.0**	**50.99 - 56.59**	**5.7**	**4.2**	**10.2**
**TSFW**	**18**	**MTP3140**	**F**	**1996**	**55.0**	**50.99 - 58.19**	**13.3**	**4.1**	**34.9**
**TSFW**	**18**	**MTP3140**	**F**	**1998**	**55.0**	**50.99 - 56.59**	**14.5**	**4.1**	**36.9**
**TSFW**	**18**	**MTP3140**	**F**	**1999**	**53.0**	**50.99 - 56.59**	**17.2**	**4.0**	**44.3**
**TSFW**	**18**	**MTP3140**	**M**	**B**	**115.3**	**113.49 - 115.29**	**15.4**	**4.0**	**35.8**
**TSFW**	**18**	**MTP3140**	**M**	**1994**	**115.3**	**113.49 - 115.29**	**11.9**	**4.3**	**31.2**
**TSFW**	**18**	**MTP3140**	**M**	**1995**	**115.3**	**113.49 - 115.29**	**10.3**	**4.2**	**27.2**
**TSFW**	**18**	**MTP3140**	**M**	**1996**	**115.3**	**113.49 - 115.29**	**12.3**	**4.3**	**34.4**
**TSFW**	**18**	**MTP3140**	**M**	**1998**	**115.3**	**113.49 - 115.29**	**16.5**	**4.1**	**40.4**
TSFW	19	SG	M	2007	38.8	31.51 - 46.21	4.1	3.7	8.7
MSFW	1	SG	F	B	23.6	19.61 - 24.41	4.9	3.7	10.3
MSFW	1	SG	F	B	25.2	18.01 - 31.21	3.8	3.7	8.0
MSFW	1	SG	F	2007	27.2	21.61 - 32.81	4.3	3.7	8.6
MSFW	1	SG	M	2006	29.1	22.61 - 34.21	5.0	3.6	10.5
MSFW	2	SG	F	B	12.0	0.01 - 27.71	4.7	3.7	12.2
MSFW	2	SG	F	2007	6.0	0.01 - 16.01	5.2	3.7	13.3
**MSFW**	**2**	**SG**	**M**	**B**	**4.0**	**0.01 - 13.01**	**6.3**	**3.7**	**15.5**
**MSFW**	**2**	**SG**	**M**	**2005**	**8.0**	**0.01 - 17.01**	**5.5**	**3.7**	**14.8**
**MSFW**	**2**	**SG**	**M**	**2007**	**7.0**	**0.01 - 15.01**	**9.1**	**3.7**	**22.9**
MSFW	5	MTP3346	F	B	5.0	0.01 - 10.21	5.3	2.5	7.3
MSFW	5	MTP3346	F	B	17.2	15.21 - 18.11	3.3	2.5	5.1
MSFW	5	MTP3346	F	2003	6.0	0.01 - 10.21	6.4	2.6	9.0

MSFW	5	MTP3346	F	2003	17.2	15.21 - 18.11	3.5	2.6	5.5
MSFW	5	MTP3346	M	2005	2.0	0.01 - 4.01	3.3	2.6	26.9
MSFW	11	MTP3234	F	2004	12.0	2.01 - 28.11	5.1	3.6	15.8
MSFW	12	MTP3346	M	B	27.8	20.81 - 32.81	2.7	2.6	5.1
MSFW	12	MTP3346	M	2003	28.8	20.81 - 32.81	2.6	2.6	4.7
MSFW	14	MTP3234	F	B	25.5	22.01 - 35.11	3.5	3.5	9.0
**MSFW**	**18**	**MTP3140**	**F**	**B**	**55.0**	**50.99 - 56.59**	**19.3**	**4.2**	**42.5**
**MSFW**	**18**	**MTP3140**	**F**	**1995**	**55.0**	**50.99 - 56.59**	**8.2**	**4.5**	**25.8**
**MSFW**	**18**	**MTP3140**	**F**	**1998**	**55.0**	**50.99 - 56.59**	**14.2**	**4.2**	**39.5**
**MSFW**	**18**	**MTP3140**	**F**	**1999**	**55.0**	**50.99 - 56.59**	**17.0**	**4.1**	**43.1**
**MSFW**	**18**	**MTP3140**	**M**	**1994**	**106.0**	**101.99 - 110.69**	**5.6**	**4.4**	**17.4**
**MSFW**	**18**	**MTP3140**	**M**	**1995**	**111.5**	**110.69 - 115.29**	**5.7**	**4.3**	**20.6**
**MSFW**	**18**	**MTP3140**	**M**	**1996**	**104.0**	**101.49 - 108.69**	**7.0**	**4.5**	**22.0**
**MSFW**	**18**	**MTP3140**	**M**	**1998**	**108.0**	**101.99 - 111.49**	**12.4**	**4.2**	**35.2**
%SDM	4	MTP3346	M	B	1.0	0.01 - 2.01	2.7	2.5	11.3
**%SDM**	**5**	**MTP3346**	**F**	**B**	**1.0**	**0.01 - 7.01**	**6.0**	**2.5**	**7.5**
**%SDM**	**5**	**MTP3346**	**F**	**2003**	**2.0**	**0.01 - 7.21**	**4.4**	**2.5**	**5.9**
**%SDM**	**5**	**MTP3346**	**F**	**2005**	**0.0**	**0.01 - 6.01**	**4.8**	**2.6**	**14.1**
%SDM	5	MTP3346	M	B	0.0	0.01 - 3.01	5.3	2.5	6.0
%SDM	5	MTP3346	M	2003	0.0	0.01 - 3.01	4.9	2.4	5.7
%SDM	14	MTP3234	M	B	27.5	16.01 - 35.51	6.1	3.7	18.0
%SDM	14	MTP3234	M	2004	18.0	14.01 - 37.51	4.0	3.7	9.9
%SDM	14	MTP3234	M	2004	27.5	14.01 - 37.51	4.1	3.7	11.9
%SDM	17	MTP3140	F	1996	31.0	17.39 - 43.09	4.6	4.4	15.7
**%SDM**	**18**	**MTP3140**	**F**	**B**	**55.0**	**50.69 - 58.19**	**17.8**	**4.2**	**41.2**
**%SDM**	**18**	**MTP3140**	**F**	**1994**	**55.0**	**50.69 - 58.19**	**6.4**	**4.4**	**22.9**
**%SDM**	**18**	**MTP3140**	**F**	**1995**	**53.0**	**46.69 - 56.59**	**8.0**	**4.3**	**27.5**
**%SDM**	**18**	**MTP3140**	**F**	**1996**	**55.0**	**50.99 - 58.19**	**5.7**	**4.4**	**25.1**
**%SDM**	**18**	**MTP3140**	**F**	**1998**	**55.0**	**50.99 - 56.59**	**13.3**	**4.1**	**37.9**
**%SDM**	**18**	**MTP3140**	**F**	**1999**	**55.0**	**50.69 - 56.59**	**13.7**	**4.1**	**37.2**
**%SDM**	**18**	**MTP3140**	**M**	**1994**	**115.3**	**113.49 - 115.29**	**9.9**	**4.4**	**33.8**
**%SDM**	**18**	**MTP3140**	**M**	**1995**	**111.5**	**107.99 - 115.29**	**8.2**	**4.8**	**29.1**
**%SDM**	**18**	**MTP3140**	**M**	**1996**	**108.0**	**101.49 - 111.49**	**5.0**	**4.5**	**20.6**
**%SDM**	**18**	**MTP3140**	**M**	**1999**	**115.3**	**113.49 - 115.29**	**13.6**	**4.3**	**36.7**

QTLs present in at least two years are shown in bold.
M: male; F: female
CI: confidence interval
MBW: mean berry weight; MSN: mean seed number; TSFW: total seed fresh weight; MSFW: mean seed fresh weight; %SDM: seed dry matter percentage; RESN: residual berry weight unexplained by seed number; RESFW: residual berry weight unexplained by total seed fresh weight
