# Supplementary material for: New stable QTLs for berry weight do not colocalize with QTLs for seed traits in cultivated grapevine (Vitis vinifera L.)
Source: BMC Plant Biol. 2013 Dec 19;13:217. doi: 10.1186/1471-2229-13-217 (PMC3878267; doi:10.1186/1471-2229-13-217)

Additional file 12: Figure S3 - Stable QTLs for seven seed and berry-related traits in the grapevine mapping population MTP3346 (consensus map). The confidence intervals (CIs) shown are for the inter-year BLUPs of the traits for which a QTL overlapping with this CI was also found in at least two different years. Distances are in Kosambi cM. Seed-related traits are in brown. MSN: mean seed number; %SDM: seed dry matter percentage.

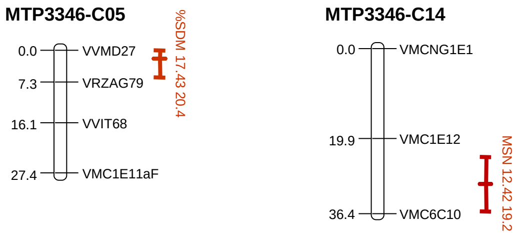

Supplement: Additional file 12: Figure S3 — Stable QTLs for seven seed and berry-related traits in the grapevine mapping population MTP3346 (consensus map). [file 1471-2229-13-217-S12.pdf]
