# Supplementary material for: New stable QTLs for berry weight do not colocalize with QTLs for seed traits in cultivated grapevine (Vitis vinifera L.)
Source: BMC Plant Biol. 2013 Dec 19;13:217. doi: 10.1186/1471-2229-13-217 (PMC3878267; doi:10.1186/1471-2229-13-217)
Supplement: Additional file 13: Figure S4 — Stable QTLs for seven seed and berry-related traits in four grapevine mapping populations (parental maps). [file 1471-2229-13-217-S13.pdf]

Additional file 13: Figure S4 - Stable QTLs for seven seed and berry-related traits in four grapevine mapping populations (parental maps). The confidence intervals (CIs) shown are for the inter-year BLUPs of the traits for which a QTL overlapping with this CI was also found in at least two different years. Distances are in Kosambi cM. Berry-related traits are in purple and seed-related traits in brown. MBW: mean berry weight; MSN: mean seed number; TSFW: total seed fresh weight; MSFW: mean seed fresh weight; %SDM: seed dry matter percentage; RESN: residual berry weight unexplained by seed number; RESFW: residual berry weight unexplained by total seed fresh weight.

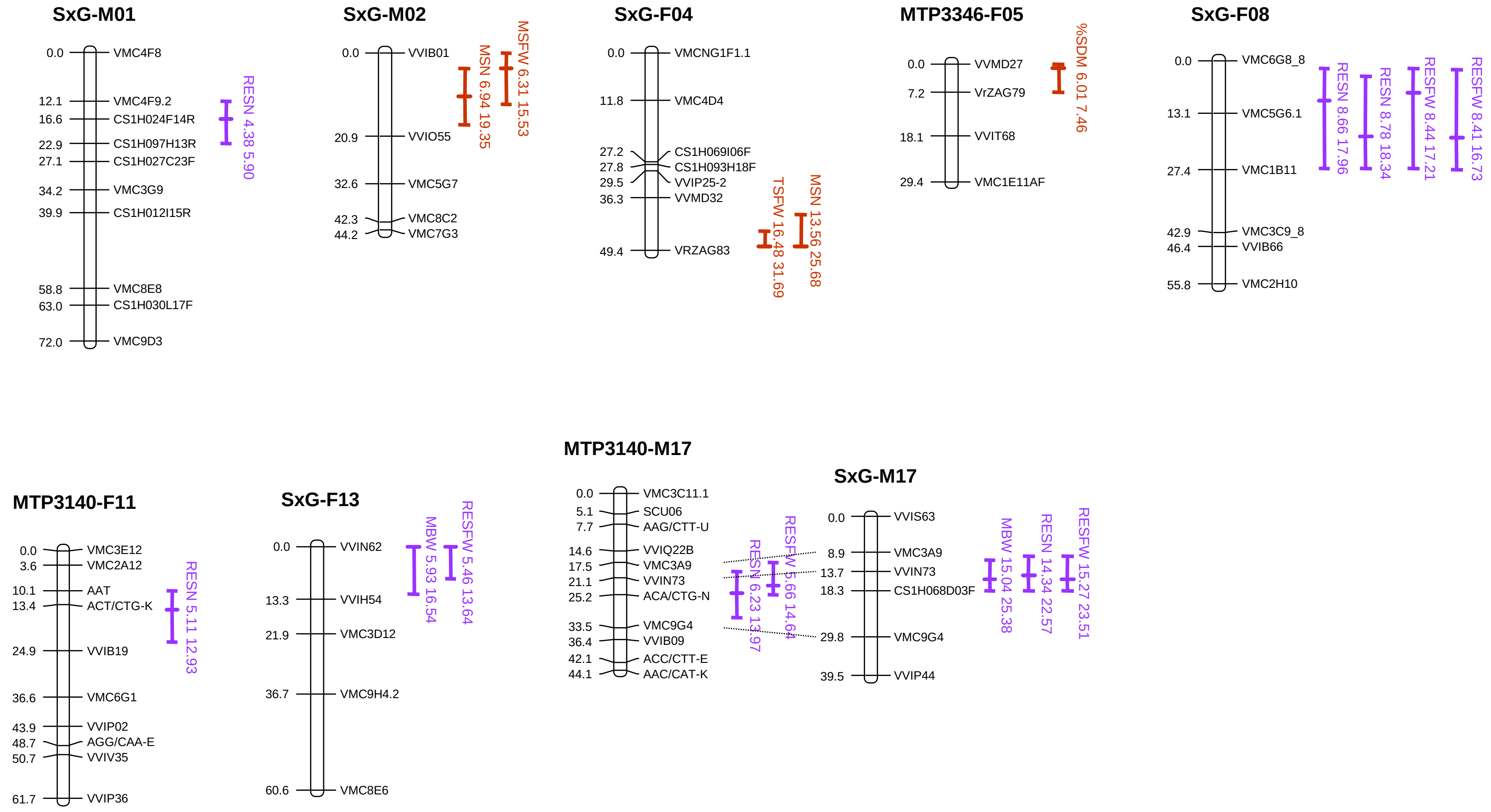

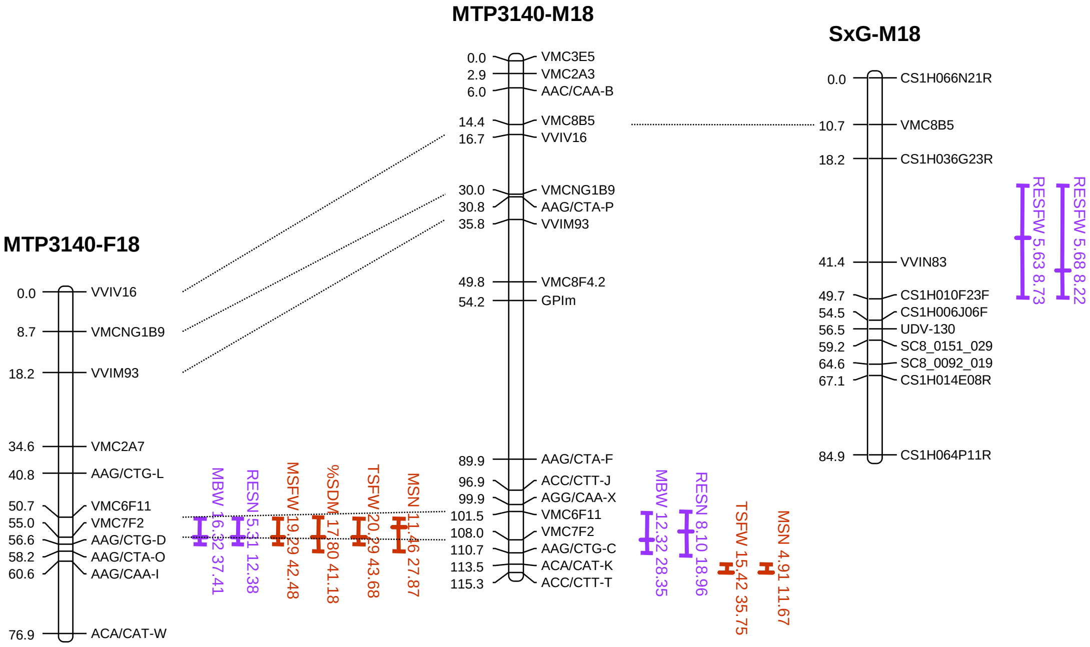
MTP3140-F18
0.0 VVIV16
8.7 VMCNG1B9
18.2 VVIM93
34.6 VMC2A7
40.8 AAG/CTG-L
50.7 VMC6F11
55.0 VMC7F2
56.6 AAG/CTG-D
58.2 AAG/CTA-O
60.6 AAG/CAA-I
76.9 ACA/CAT-W
MBW 16.32 37.41
RESN 5.31 12.38
MSFW 19.29 42.48
%SDM 17.80 41.18
TSFW 20.29 43.68
MSN 11.46 27.87
MTP3140-M18
0.0 VMC3E5
2.9 VMC2A3
6.0 AAC/CAA-B
14.4 VMC8B5
16.7 VVIV16
30.0 VMCNG1B9
30.8 AAG/CTA-P
35.8 VVIM93
49.8 VMC8F4.2
54.2 GPIm
89.9 AAG/CTA-F
96.9 ACC/CTT-J
99.9 AGG/CAA-X
101.5 VMC6F11
108.0 VMC7F2
110.7 AAG/CTG-C
113.5 ACA/CAT-K
115.3 ACC/CTT-T
MBW 12.32 28.35
RESN 8.10 18.96
TSFW 15.42 35.75
MSN 4.91 11.67
SxG-M18
0.0 CS1H066N21R
10.7 VMC8B5
18.2 CS1H036G23R
41.4 VVIN83
49.7 CS1H010F23F
54.5 CS1H006J06F
56.5 UDV-130
59.2 SC8_0151_029
64.6 SC8_0092_019
67.1 CS1H014E08R
84.9 CS1H064P11R
RESFW 5.63 8.73
RESFW 5.68 8.22
