# Supplementary material for: New stable QTLs for berry weight do not colocalize with QTLs for seed traits in cultivated grapevine (Vitis vinifera L.)
Source: BMC Plant Biol. 2013 Dec 19;13:217. doi: 10.1186/1471-2229-13-217 (PMC3878267; doi:10.1186/1471-2229-13-217)
Supplement: Additional file 14: Table S10 — Intervals explored and number of positional candidate genes found. [file 1471-2229-13-217-S14.pdf]

Additional file 14: Table S10 - Intervals explored and number of positional candidate genes found.

LG	Trait(s)	Population	Left marker [1]	Right marker [1]	Left limit of the interval searched (bp)	Right limit of the interval searched (bp)	Number of genes found[3]
1	MSFW	SxG	VMC4F9-2	VMC3G9	3,694,116	5,268,843	127
2	MSN	SxG	VVIB01	VVIO55	3,690,461	4,584,558	87
2	MSFW	SxG	VVIB01	VVIO55	3,392,429	4,286,526	77
4	TSFW	SxG	VRZAG83	-	19,612,664	20,908,676	124
4	MSN	SxG	VVMD32	VRZAG83	19,115,859	20,411,871	120
5	%SDM	MTP3346	VVMD27	VVIT68	4,308,971	5,288,353	81
8	MBW - RESFW	SxG	VMC5G6-1	VMC1B11	12,396,132	13,529,508	94
8	RESN	SxG	VMC1B11	VMC3C9	15,353,475	16,479,716	101
11	MBW - RESN	MTP3140	VVS2	VVIB19	3,758,521	4,667,573	79
12	MSFW	MTP3140	VMC8G6	VMC2H4	4,789,395	6,105,667	107
13	RESFW	SxG	VVIH54	VMC3D12	3,713,672	5,993,789	167
14	%SDM	MTP3234	VMC2H12	VMC2H5	14,752,927	20,091,576	131
14	MSN	MTP3346	VMC1E12	VMC6C10	13,495,265	18,945,265	117
17	MBW	MTP3140	VVIN73	VMC9G4	5,132,528	6,850,126	150
17	RESN	MTP3140	VVIN73	VMC9G4	4,645,875	6,363,473	136
17	MBW - RESN - RESFW	SxG	VVIN73	VMC9G4	5,256,427	6,745,907	126
18	MBW - TSFW - %SDM - MSFW	MTP3140	VVMD17	VMC7F2	26,372,842	28,102,527[2]	100
18	MSN	MTP3140	VVMD17	VMC7F2	26,530,086	28,941,162[2]	147
18	RESN	MTP3140	VVMD17	VMC7F2	24,590,742	27,735,624	165
18	MBW - RESN	SxG	VVIN83	VVIU04	10,416,561	11,910,931	158
18	RESFW	SxG	VVIN83	VVIU04	10,914,685	12,409,054	150

[1] Nearest marker present on the 12X genome sequence

[2] Upper limit of the CI lower than LOD peak position + 3 cM

MBW: mean berry weight; MSN: mean seed number; TSFW: total seed fresh weight; MSFW: mean seed fresh weight; %SDM: seed dry matter percentage; RESN: residual berry weight unexplained by seed number; RESFW: residual berry weight unexplained by total seed fresh weight

[3] Genoscope 12X annotation
