# Supplementary material for: New stable QTLs for berry weight do not colocalize with QTLs for seed traits in cultivated grapevine (Vitis vinifera L.)
Source: BMC Plant Biol. 2013 Dec 19;13:217. doi: 10.1186/1471-2229-13-217 (PMC3878267; doi:10.1186/1471-2229-13-217)
Supplement: Additional file 15: Table S11 — Published functional candidate genes potentially involved in seed and/or berry development not colocalized with QTLs detected in the present study [92-106]. [file 1471-2229-13-217-S15.pdf]

Additional file 15: Table S11 - Published functional candidate genes potentially involved in seed and/or berry development not colocalized with QTLs detected in the present study. Gene numbers were obtained from the automatic annotation provided with the Genoscope 12X whole genome sequence release of PN40024 (http://www.genoscope.cns.fr/externe/GenomeBrowser/Vitis/; [1]).

Grapevine 12X gene ID	Gene name	LG	position in 12X	putative homologous gene and/or function	Reference
GSVIVT01012249001	*VvSEP4*	1	194107 .. 206538	*MIKC* gene expressed in flowers and fruit	[92]
GSVIVT01012231001	-	1	381267 .. 382921	Cytochrome P450 78A-like protein in *Solanum lycopersicum* (tomato), harboring the causal SNP of a berry weight QTL	[56]
GSVIVT01012110001	*VvBS2*	1	1353272 .. 1355920	*MIKC* gene expressed in flowers and fruit	[92]
GSVIVT01011926001	-	1	2954243..2955469	ZmCNR2, involved in tissue growth activity in maize	[59]
GSVIVG01020033001	*VvHB13*	1	11304894 .. 11307266	*Homeo Box 13* (*Arabidopsis*), homeotic protein	[89]
GSVIVT01020031001	*VvSUC12*	1	11312668 .. 11322931	Proton-sucrose symporter (cDNA cloned in *V. vinifera* and upregulated in berries at *véraison*)	[93]
GSVIVG01010521001	*VvMADS4*	1	21368898 .. 21386389	*Agamous like 9 - Sepallata 3* (*Arabidopsis*) MADS-box flower and berry development	[89, 90]
GSVIVT01010521001	*VvSEP3=MADS4*	1	21368898 .. 21386389	*MIKC* gene expressed in fruits with increasing expression from flowers to fruits	[90]
GSVIVT01004930001	*VvCEB1*	1	21582290 .. 21584461	Berry-specific basic helix-loop-heliw transcription factor affecting cell size	[94]
GSVIVT01001124001	*VvXET2*	1	22826373 .. 22828816	Xyloglucan endotransglycosylase (cell-wall modifying enzyme expressed during berry development)	[78]
GSVIVT01001124001	*XTH*	1	22826373 .. 22828816	Xyloglucan:xyloglucosyl transferase involved in ethylene-induced berry expansion	[11]
GSVIVT01019523001	*CEL*	2	1331283 .. 1335833	Cellulase similar to endo-beta-(1,4)-glucanase with expression linked to berry development	[76]
GSVIVT01019653001	-	2	2231724..2233589	Cytochrome P450, family 78, subfamily A, polypeptide 9 (Arabidopsis), controlling fruit size	[95]

GSVIVG01019761001	*VvJMJ*[1]	2	3074405 .. 3119122	*F2K11.14*, transcription factor jumonji, associated with berry weight and length	[45, 89]
GSVIVT01023919001	*EXPL*	3	2486948 .. 2488738	Expansin *EXPL2* with expression linked to berry development	[76]
GSVIVG01023852001	*VvHMGR3*	3	2970316 .. 2972915	*HMG1* (Arabidopsis), HMGcoA reductase involved in cell elongation and isoprenoid synthesis	[96]
GSVIVG01031753001	*VvFw2.2.2*	3	4060722 .. 4066802	*Fw2.2* (tomato), negative regulator of cell division in fruit	[58]
GSVIVG01031754001	*VvFw2.2.1*	3	4067224 .. 4070468	*Fw2.2* (tomato), negative regulator of cell division in fruit	[58]
GSVIVG01031755001	*VvFw2.2.3*	3	4070578 .. 4072718	*Fw2.2* (tomato), negative regulator of cell division in fruit	[58]
GSVIVG01031941001	*VvBURP1*	3	5579524 .. 5580226	*Unknown seed protein like 1* (Arabidopsis), seed development	[89]
GSVIVG01031943001	*VvSVP2*	3	5581839 .. 5591761	-	[89]
GSVIVT01034945001	*VvGA2ox4*	5	343990 .. 346779	GA2-oxidase expressed in berry	[97]
GSVIVT01027774001	*Grip 3 = Grip 4*	5	7580440 .. 7581363	Proline-rich cell wall protein	[98]
GSVIVT01027789001	*Grip 15*	5	7787811 .. 7788943	Proline-rich cell wall protein	[98]
GSVIVT01027790001	*Grip 13*	5	7792153 .. 7793327	Proline-rich cell wall protein	[98]
GSVIVT01029048001	*VvPL1*	5	11376715 .. 11380856	Pectate lyase (cell-wall modifying enzyme expressed during berry development)	[78]
GSVIVT01031593001	*VvXET1*	5	18634353 .. 18636084	Xyloglucan endotransglycosylase (cell-wall modifying enzyme expressed during berry development)	[78]
GSVIVG01037533001	*VvYABBY2*	6	11951270 .. 11958015	*Fasciated* (tomato), controls carpel number	[89]
GSVIVT01031486001	*XET*	6	18028143 .. 18029883	Xyloglucan:xyloglucosyl transferase with expression linked to berry development	[76]

GSVIVT01031464001	-	6	18257605 .. 18258772	Major intrinsic family protein involved in ethylene-induced berry expansion	[11]
GSVIVG01031404001	*VvHD2C*	6	19041783 .. 19047544	*HD2C*, histone deacetylase 2C	[89]
GSVIVT01028281001 ?	-	7	5457683 .. 5458417 ?	Integrase (EST expressed only after GA treatment)	[99]
GSVIVG01033891001	*VvSPY*	8	16964462 .. 16984299	*SPINDLY* (Arabidopsis), repressor of GA and positive regulation of cytokinin signalling	[100]
GSVIVG01033743001	*VvRPL24*	8	18192061 .. 18195774	*Ribosomal protein like 24* (Arabidopsis), auxin signaling and gynoecium development	[101]
GSVIVT01033540001	*BG*	8	19873175 .. 19874681	Glycosyl hydrolase family protein, similar to beta-(1,3)-glucanase, with expression linked to berry development	[76]
GSVIVT01033303001	*VvPG1*	8	21672956 .. 21675817	polygalacturonase (cell-wall modifying enzyme expressed during berry development), involved in ethylene-induced berry expansion	[11, 78]
GSVIVT01033253001	*VvAGL15.2*	8	22162819 .. 22165051	*MIKC* gene expressed in flowers and fruit	[92]
GSVIVT01017211001	*VvGA3ox4*	9	5453528 .. 5458751	GA3beta-hydrolase gene expressed in berry	[97]
GSVIVT01017353001	-	9	7092507 .. 7134534	*CHY1* (EST expressed only after GA treatment)	[99]
GSVIVT01021303001	*VvAG2*	10	3730147 .. 3738385	*MIKC* gene expressed in flowers and fruit	[92]
GSVIVT01021445001	*SIRK*	10	5451220 .. 5456608	K + channel protein (EST expressed only after GA treatment)	[99]
GSVIVT01021468001	*VvGA2ox2*	10	5846741 .. 5848388	GA2-oxidase expressed in berry	[97]
GSVIVT01000802001	*VvAG1=MADS1*	12	279315 .. 292144	*MIKC* gene expressed in flowers and fruit (Agamous Arabidopsis, MADS box fruit development)	[102]
GSVIVT01023135001	*VvPME1*	12	22226001 .. 22228636	Pectin methylesterase (cell-wall modifying enzyme expressed during berry development)	[76, 78]
GSVIVT01032681001	*Vlexp-2= Vlexp-3*	13	1578645 .. 1580497	Expansin (cDNA cloned in *V. vinifera* with expression related to ripening)	[103]

GSVIVT01001437001	*VvAGL15.1*	13	20980100 .. 20988849	*MIKC* gene expressed in flowers and fruit	[92]
GSVIVG01036549001	*VvMADS6*	14	23320331 .. 23341234	*Apetala 1* (Arabidopsis), MADS box flower development	L. Torregrosa (pers. com.)
GSVIVT01036551001	*VvSEP1=MADS2*	14	23363222 .. 23379548	*MIKC* gene expressed in flowers and fruit	[90]
GSVIVT01032383001	-	14	26847692 .. 26853716	Pseudouridine synthase/transporter (EST expressed only after GA treatment)	[99]
GSVIVT01011354001	-	14	28912919 .. 28915800	Serine carboxypeptidase-like 44 (EST expressed only after GA treatment)	[99]
GSVIVT01018185001	-	15	13453328..13458913	PavCNR12, Prunus avium cell number regulator 12 (CNR12) gene, most likely underlying a fruit size QTL in sweet cherry	[55]
GSVIVT01027577001	*VvAGL6=MADS3*	15	15406740 .. 15424492	*MIKC* gene expressed in flowers and fruit (Agl6 and Agl13 Arabidopsis, MADS box seed development)	[90]
GSVIVT01027404001	-	15	17005434..17007131	Cytochrome P450, family 78, subfamily A, polypeptide 9 (Arabidopsis), controlling fruit size	[95]
GSVIVT01026942001	*AQ3=PIP1;2*	15	19199215 .. 19206926	Plasma membrane intrinsic protein 1A (*PIP1A*); aquaporin *PIP1.1* with expression linked to berry development	[76]
GSVIVT01024235001	*AQUA*	16	102222 .. 103573	Aquaporin TIP3-2 (Arabidopsis), major intrinsic family protein, involved in ethylene-induced berry expansion	[11]
GSVIVT01018665001	-	16	11481264..11488666	PavCNR12, Prunus avium cell number regulator 12 (CNR12) gene, most likely underlying a fruit size QTL in sweet cherry	[55]
GSVIVT01018599001	*Grip 28*	16	12503456 .. 12517626	Ripening-related protein, cell wall	[98]
GSVIVT01018453001	*VvGA20ox1*	16	14861395 .. 14863190	GA20-oxidase expressed in berry	[97]
GSVIVT01007582001	*PL*	17	11674724 .. 11676881	Pectate lyase family protein with expression linked to berry development	[76]
GSVIVT01012906001	*S27a*	17	13268008 .. 13273528	Ubiquitin extension protein	[104]

GSVIVT01029411001	*EX = Vlexp-1*	17	16771613 .. 16773967	Expansin with expression related to ripening, involved in ethylene-induced berry expansion	[11, 103]
GSVIVT01013458001	-	18	307575 .. 308424	Uncharacterized, significantly associated with berry size	[44]
GSVIVG01013435001	*VvHMGR1*	18	494023 .. 498427	*HMG1* (Arabidopsis), HMGcoA reductase involved in cell elongation and isoprenoid synthesis, associated with berry length	[45, 96]
GSVIVT01013402001	-	18	686115 .. 690591	Endoribonuclease/protein kinase lre1p-like (Arabidopsis), significantly associated with berry size	[44]
GSVIVT01013367001	-	18	927341 .. 932130	Catalase, significantly associated with berry size	[44]
GSVIVT01013363001	-	18	949015 .. 961356	Ribosome biogenesis, significantly associated with berry size	[44]
GSVIVT01008762001	*EXP1*	18	1778795 .. 1780353	Expansin *EXP11* with expression linked to berry development	[76]
GSVIVT01008762001	*AQ1*	18	1778795 .. 1780353	Major intrinsic family protein MIP with expression linked to berry development	[76]
GSVIVG01008806001	*VvMADS9*	18	2291789 .. 2294142	*Pistilata* (Arabidopsis), MADS box flower development	[89]
GSVIVT01009024001	*VvHT2*	18	4363667 .. 4369067	Hexose transporter (cDNA cloned in *V. vinifera* with increased expression around *véraison*)	[105]
GSVIVG01009171001	*VvAGL22.2*	18	5694493 .. 5711399	*Agamous like 22* (Arabidopsis), MADS box flower development	L. Torregrosa (pers. com.)
GSVIVT01009219001	*VvAGL17.1*	18	6350901 .. 6376307	*Agamous like 17* (Arabidopsis), MADS box	[92]
GSVIVT01009254001	*VvSUC11*	18	6693573 .. 6703325	Proton-sucrose symporter (cDNA cloned in *V. vinifera* and upregulated in berries at *véraison*)	[93]
-	*VvKO*	18	9625800 .. 9625486 (blast)	Ent-Kaurene oxidase expressed in berry	[97]
GSVIVT01005051001	*VvCBP1*	Un	17696019 .. 17698346	Calcium-binding protein involved in embryo	[106]

				development	
- (AJ237987)	*Grip 68*	Un	94441 .. 154135	Ripening-related protein, cell wall	[98]
GSVIVT01007333001	*CS*	Un	31413744 .. 31415587	Cellulose synthase family protein involved in ethylene-induced berry expansion	[11]

[1] Not a berry or seed QTL because sex QTL
